# The Formation of Multi-synaptic Connections by the Interaction of Synaptic and Structural Plasticity and Their Functional Consequences

**DOI:** 10.1371/journal.pcbi.1004031

**Published:** 2015-01-15

**Authors:** Michael Fauth, Florentin Wörgötter, Christian Tetzlaff

**Affiliations:** Georg-August University Göttingen, Third Institute of Physics, Bernstein Center for Computational Neuroscience, Göttingen, Germany; Duke University, UNITED STATES

## Abstract

Cortical connectivity emerges from the permanent interaction between neuronal activity and synaptic as well as structural plasticity. An important experimentally observed feature of this connectivity is the distribution of the number of synapses from one neuron to another, which has been measured in several cortical layers. All of these distributions are bimodal with one peak at zero and a second one at a small number (3–8) of synapses.

In this study, using a probabilistic model of structural plasticity, which depends on the synaptic weights, we explore how these distributions can emerge and which functional consequences they have.

We find that bimodal distributions arise generically from the interaction of structural plasticity with synaptic plasticity rules that fulfill the following biological realistic constraints: First, the synaptic weights have to grow with the postsynaptic activity. Second, this growth curve and/or the input-output relation of the postsynaptic neuron have to change sub-linearly (negative curvature). As most neurons show such input-output-relations, these constraints can be fulfilled by many biological reasonable systems.

Given such a system, we show that the different activities, which can explain the layer-specific distributions, correspond to experimentally observed activities.

Considering these activities as working point of the system and varying the pre- or postsynaptic stimulation reveals a hysteresis in the number of synapses. As a consequence of this, the connectivity between two neurons can be controlled by activity but is also safeguarded against overly fast changes.

These results indicate that the complex dynamics between activity and plasticity will, already between a pair of neurons, induce a variety of possible stable synaptic distributions, which could support memory mechanisms.

## Author Summary

The connectivity between neurons is modified by different mechanisms. On a time scale of minutes to hours one finds synaptic plasticity, whereas mechanisms for structural changes at axons or dendrites may take days. One main factor determining structural changes is the weight of a connection, which, in turn, is adapted by synaptic plasticity. Both mechanisms, synaptic and structural plasticity, are influenced and determined by the activity pattern in the network. Hence, it is important to understand how activity and the different plasticity mechanisms influence each other. Especially how activity influences rewiring in adult networks is still an open question.

We present a model, which captures these complex interactions by abstracting structural plasticity with weight-dependent probabilities. This allows for calculating the distribution of the number of synapses between two neurons analytically. We report that biologically realistic connection patterns for different cortical layers generically arise with synaptic plasticity rules in which the synaptic weights grow with postsynaptic activity. The connectivity patterns also lead to different activity levels resembling those found in the different cortical layers. Interestingly such a system exhibits a hysteresis by which connections remain stable longer than expected, which may add to the stability of information storage in the network.

## Introduction

The connectivity between neurons - i.e., the number of synapses and their transmission efficiencies (weights) - determines information processing and storage in neural networks and, thus, also the cortex. Thus, in order to understand the functionality of cortical neural networks, we have to understand how they generate their connectivity.

Generally, there are two major processes capable of connectivity changes: the first process, so-called structural or architectural plasticity, builds and removes synapses between neurons [1–4]. The transmission efficiency of these synapses is, in turn, modified by the second process named synaptic plasticity [5–7].

Synaptic plasticity was first postulated by Donald O. Hebb [5]. Later on, experiments showed persistent strengthening and weakening of the synaptic transmission efficacy due to neuronal activity [6,8,9]. Besides firing frequencies, different timings of pre- and postsynaptic action potentials play an important role [7,10]. On a longer time scale, it has also been shown, that synaptic weights can be homoeostatically regulated to reach a certain firing frequency in the network [11].

Structural plasticity, on the one hand, refers to the outgrowth and retraction of axons and dendrites, which is primarily taking place during developmental phases or after major injuries of the network structure [1]. On the other hand, it refers to the process of creating and removing synapses, which is the predominant process in adult networks [12]. As the majority of cortical synapses resides on so-called dendritic spines, we can investigate their dynamics to get an intuition about the dynamics of synapses. Spines are highly motile structures which can appear and disappear on a time scale of hours to days. Their lifetime has been shown to depend on their head-volume [3, 12, 13]. Furthermore, this head-volume correlates with the strength of the excitatory postsynaptic potentials (EPSPs) from the corresponding synapse [14] - the electro-physiological equivalent of the synaptic weight. It has also been shown that stimulation protocols which potentiate or depress the synaptic weight enlarge [15] or shrink [16] the spine head respectively. Thus, synaptic plasticity influences the spine head volume, which determines the probability of structural changes. This indicates a strong interaction of synaptic and structural plasticity.

A recent long-term *in vitro* study revealed that the dynamics of the spine volume, and, therefore, the removal of synapses, can be treated as a random process on longer time scales [13]. Furthermore, this study confirms the influence of synaptic plasticity mechanisms on this random process.

Therefore, the emergence of neuronal connectivity should be understandable from the interaction between synaptic and structural plasticity. To explore this interaction experimentally, the changes of the weights of synapses would have to be monitored on the time scale of structural changes [17]. Unfortunately experiments have not been extended to this time scale yet.

Thus, as the two processes cannot be measured simultaneously, their interaction currently can only be investigated by theoretical models. For both processes there are already plenty of commonly used mathematical formulations: Synaptic plasticity has been modelled, e.g., by the Hebbian rule [18], the Oja rule [19], the Bienenstock-Cooper-Munro rule [18, 20], or even by arbitrary polynomials in pre- and postsynaptic activity and the synaptic weight [21] for rate-based systems. Further on, there is also a variety of spike-timing-dependent plasticity rules (see, e.g., [22–24]). Mechanisms, which prevent divergence of weights, have been proposed for both classes [18, 25]. On the other hand, there are also several structural plasticity models: Some of them describe the activity-dependent restructuring of networks during development or after major injuries [26–28], whereas others tried to describe connection changes from an information theoretic point of view [29, 30].

The interaction of synaptic and structural plasticity has mostly been addressed by simulation studies [31–33]. These studies already showed the importance of structural plasticity to generate certain statistical network features (e.g., network motifs [34, 35]), but provide little analytical understanding how these features are generated.

**Figure 1:**
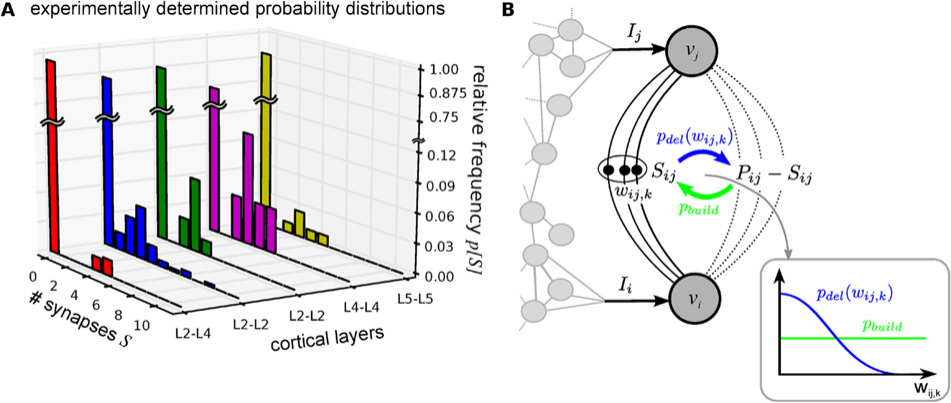
Experimentally obtained distributions of the number of synapses on a single connection and the here proposed structural plasticity model. *(A)* The relative frequencies *p*[*S*] of the number of synapses between two neurons obtained from experiments [10, 36–39] show strong peaks for no synapses and 3–8 synapses. The probability for no synapses (*S* = 0) reaches from 0.75 to 0.99 (for [38] this probability was taken from [81]). *(B)* Scheme of the proposed structural plasticity model shows relevant quantities of the model for one connection. Synapses are created from a pool of potential synapses *P*_*ij*_ with a constant probability *p*_*build*_. Each of the *S*_*ij*_ realised synapses has a weight *w*_*ij*,*k*_ (*k* = 1 … *S*_*ij*_) which develops according to a synaptic plasticity rule. Synapse removal happens with a weight-dependent probability *p*_*del*_(*w*_*ij*,*k*_). All influences which do not relate to the examined neurons are modelled by currents *I*_*i*_ or *I*_*j*_. *Inset:* An example for the weight-dependency of building and removal probabilities shows that smaller synapses are more likely to be deleted. (See main text for more details)

In order to obtain a better analytical understanding, a two neuron system and the probability distribution of the number of synapses from one neuron to the other is used as a benchmark, because it has been measured experimentally. In those experiments, the number of synapses between two neurons is estimated by quantal analysis of the excitatory postsynaptic potentials from patch clamp experiments [10, 36–39]. The distributions of the number of synapses between two neurons obtained by this method typically peak between three and eight synapses and shows very low probabilities for one or two synapses (Fig. 1A). The fraction of unconnected neuron pairs in those experiments reaches from 75%−99%, such that the distribution has a second very large peak at zero synapses. Due to this large number of unconnected pairs, the connected part of the experimental distributions is based on very few (10–50) data points resulting to large statistical errors (see Text S1). Thus, these experimental data should be interpreted qualitatively rather than quantitatively.

In a first model, which was proposed to understand how these distributions emerge [40], the synapses of a connection are generated from a pool of potential contacts with a certain probability. Thereby, the probability for a certain number of potential synapses was estimated from morphological reconstructed neurons. Nevertheless, morphology on its own was not sufficient to explain the distribution of the actual number of synapses. It turned out that the probability of small numbers of synapses is strongly suppressed, which means that synapses are not created independently but rather in a collective manner. However, newly established connections typically have less synapses than old ones [3]. Thus, there must be an underlying dynamic process, which influences the stability of synapses, such that their creation appears collective on a longer time scale. The stabilisation of spines *in vivo* depends on experience-dependent (synaptic) plasticity [41, 42]. As the collective-formation model does not include activities or activity-dependent plasticity, it describes synapse formation only in a coarser way. A model which includes the interaction of synaptic and structural plasticity should provide more insight into the dynamics that lead to the shape of the experimental distribution (Fig. 1A).

In fact, another theoretical study already demonstrated [43] that these experimental distributions can be reproduced with a structural plasticity rule, which is modulated by a synaptic-plasticity-related quantity. Particularly, the authors of this study assume stochastic changes between three possible synaptic states (absent, silent or active) with probabilities modulated by a quantity which resembles an abstract version of spike-timing-dependent plasticity. Hereby, the influence of an active synapse on the postsynaptic neuron is assumed to be constant and not coupled to this plasticity-related quantity. Although this study already shows that this specific interaction of structural and synaptic plasticity can in fact yield biologically reasonable connectivity, important questions are still open: First, under the broad variety of existing synaptic plasticity rules [5, 17, 20, 21, 44], which class of learning rules can exhibit the experimentally observed behaviour in combination with stochastic structural plasticity? Second, the above discussed study [43] assumes fixed firing frequencies, although the firing frequencies between the different experimental locations differ [45–47] and, thus, may influence the structural changes. Moreover, activity levels possibly act as control signal, which drives connectivity to change in a specific way, such that it exhibits certain non-random features (see [34, 35, 40]). Therefore, it is important to evaluate, if and how changes in activity influence the connectivity.

To tackle these questions, in this study, we propose a stochastic model for structural plasticity based on weight-dependent probabilities. Using this model, we calculate the stationary probability distribution for the number of synapses between two neurons. By means of a simple approximation, we derive model-constraints under which this probability distribution corresponds to the biological measured distributions. Interestingly, one of these constraints requires the synaptic weight to grow with the postsynaptic activity, which corresponds to the Hebbian idea of synaptic plasticity. Then, we show that one example system, which matches these constraints, can explain the experimentally observed distributions from different cortical layers by different neuronal activities, which relates well to experimental observations. Considering these suitable activities as a working point of the system, we explore its activity-dependent dynamics around this working point. We find, that increasing and decreasing activity does in fact influence the connectivity strongly. More precisely, the number of synapses undergoes a hysteresis loop when changing either pre- or postsynaptic activity, which suggests potential for memory storage.

## Results

### Model of stochastic structural plasticity

In the following, we present a biologically plausible mathematical model with which the (long-term) interaction between synaptic and structural plasticity is investigated. In this model, the process with the slowest time scale is structural plasticity, which operates from days to weeks (see, e.g., [48–50]). We assume that on this time scale the spiking-dynamics of the neurons, which take place in several milliseconds, need not to be modelled explicitly. Therefore, we use rate-based neuron models. The firing frequency *v*_*i*_ of such a neuron *i* is determined by the input-output-relation *F*, which is an increasing function of the inflowing current:

$$v_{i}=F\left[\sum_{j}\sum_{k=1}^{S_{ij}}w_{ij,k}v_{j}+I_{i}\right].$$

The first component of this current are signals, which are transmitted from other neurons via synapses and calculated as the product of the presynaptic neurons’ firing frequencies *v*_*j*_ and the synaptic weights *w*_*ij*,*k*_ of the *k*th synapse from *j* to *i*: *w*_*ij*,*k*_ *v*_*j*_. Hereby, *S*_*ij*_ denotes the number of synapses from neuron *j* to neuron *i*. The second component *I*_*i*_ represents other neuron-specific influences such as leakage currents, inhibitory inputs or inputs from other cortical layers or brain areas (e.g., thalamus). Note, for better readability in this paper we do not indicate the time-dependence of activities, weights, number of synapses or stimulations. To eliminate covariant model parameters, we normalize the activities *v*_*i*_ to the interval [0, 1]. Due to the diversity of experimentally measured input-output-relations we use the logistic function *F*[*x*] = 1/ (1 + exp(−*x*)), if not stated differently. This function is both analytically tractable and it includes the transition from convex to concave input-output relation. This transition is a common feature of most biological neurons, such that we can make qualitative predictions.

As already mentioned above, to model structural plasticity, the morphology of the two neurons and their dendritic and axonal trees is abstracted to a number of potential synapses *P*_*ij*_ [51], where each potential synapse represents a location, where a dendritic spine can bridge the gap between axon and dendrite. If multiple spines can do this close to each other (clustered spines [52]), each of them is counted as a potential synapse. Thus, in the following, a *connection* between two neurons is described by the number of potential synapses *P*_*ij*_, the number of realised synapses *S*_*ij*_ and their weights *w*_*ij*,*k*_ (Fig. 1B).

At each of the *P*_*ij*_ − *S*_*ij*_ locations, where no synapse exists, a synapse can be formed with a constant probability *p*_*build*_ (formation rate). If a synapse already exists, its weight is adapted by a synaptic plasticity rule. The mathematical formulation of this rule should only depend on local quantities of the synapse: pre- and postsynaptic activity and the synaptic weight itself [21]. Furthermore, it is required that the rule leads to stable (bounded) weights $w_{ij}^{*}[v_{i},v_{j}]$ (also called fixed weights) for given pre- and postsynaptic activities.

The removal of an existing synapse can also happen with a certain probability *p*_*del*_. Inspired by biological weight-volume correlation [14] and the volume-lifetime-dependency [3, 12, 13], we model this probability as a decreasing function of the synaptic weight resulting from the plasticity rule (Fig. 1B, inset):

$$p_{del}\left[w_{ij}\right]=p_{build}^{\rho }\exp\left(-a^{2}w_{ij}^{4/3}\right)$$ (1)

where *ρ* scales the maximum deletion probability relative to the building probability and, thereby, determines whether deletion happens faster or slower than build-up, and *a* determines the influence of the weight. The exponent 4/3 has been inspired from spine-volume-dynamics from [13]. However, the results presented below, are insensitive to the exact mathematical form of this function (see Text S5).

### Equilibrium-distributions of the number of synapses on a single connection

We now investigate which connectivity emerges from the model above. Therefore, we calculate the probability *p*[*S*] that one single plastic connection from neuron *j* to neuron *i* has *S* synapses in the long-term equilibrium (rest of the network fixed; Fig. 1B). The resulting distribution is especially interesting, because it can be compared to experimental results ([10, 36–39], Fig. 1A). Furthermore, it can be calculated analytically, which we show in the following.

To describe one single plastic connection from neuron *j* to neuron *i*, the currents from all neurons other than *i* and *j* can be included into *I*_*i*_. The only current which influences the connection results from the presynaptic activity *v*_*j*_. Note, this effective *I*_*i*_ is now specific for the single connection from *j* to *i*. Multiple connections on the same postsynaptic neuron would have different values for *I*_*i*_.

In the following, some indices will be omitted for better readability: *P* := *P*_*ij*_, *S* := *S*_*ij*_ and *I* := *I*_*i*_. The influence *I*_*j*_ will not be used as it is completely determined by *v*_*j*_ = *F*(*I*_*j*_).

For given postsynaptic influence *I* and presynaptic activity *v*_*j*_, we can now calculate the equilibrium probability distribution of the number of synapses *p*[*S*] on this connection in the following way: We assume that after each structural change all weights *w*_*ij*,*k*_ converge to their fixed point before the next structural change takes place. Thus, we separate the time scales of both plasticity mechanisms as the time scale of structural changes is much longer than the one for synaptic changes [48] which has been similarly applied in [43]. Thus, for a fixed *S*, we can calculate the fixed weights $w_{ij}^{*}[v_{i}^{*}[S],v_{j}]$ and activities $v_{i}^{*}[S]$. Knowing the fixed weight for *S*, we can calculate the deletion probability for *S* synapses. Thereby, deletion probability $p_{del}(w_{ij}^{*}[v_{i}^{*}[S],v_{j}])$ and building probability *p*_*build*_ only depend on the current number of synapses but not on past values. The system can thus be treated as Markov-process with the number of synapses as states. For each of those states, we can calculate the probability to jump to any other state from the deletion and building probability and, thereby, create a transition matrix. The long-term equilibrium distribution of the number of synapses on the plastic connection (stationary distribution of the Markov-process) can now be calculated from the (dominant) eigenvectors of the transition matrix.

However, this requires solving a system of equations and is not expressible by a simple formula, which would allow us to investigate the influence of the different components of our model. Thus, to approach the shape of this distribution in a more analytical way, the so-called *first-step-approximation* can be used [43]. In this approximation the system is only allowed to create or remove one synapse at one time step. Then, the probability flow between two neighbouring states *S* − 1 and *S* is in equilibrium given by:

p[(S−1)→S]=p[S→(S−1)](detailed balance)

$$\text{with}\;p[(S-1)\to S]=\;\underset{\text{state probability}}{\underset{︸}{p[S-1]}}\;\cdot \;\underset{\text{transition probability}}{\underset{︸}{(P-S+1)\cdot p_{build}}}$$

$$p[S\to (S-1)]=\;\underset{\text{state probability}}{\underset{︸}{p[S]}}\;\cdot \;\underset{\text{transition probability}}{\underset{︸}{S\cdot p_{del}\left[w_{ij}^{*}\left[v_{i}[S],v_{j}\right]\right]}}.$$

From this we deduce the ratio of the probabilities between two neighbouring states in equilibrium

$$\Delta_{p}[S]:=\frac{p[S]}{p[S-1]}=\frac{\left(P-S+1\right)}{S}\frac{p_{build}}{p_{del}\left[w_{ij}^{*}[v_{i}[S],v_{j}]\right]}.$$ (2)

By using these ratios, the whole distribution can be recursively calculated as multiples of *p*[*S* = 0], which can then be derived from the requirement that the probability distribution sums up to one (see Eq. 7). This provides us with a formula to calculate *p*[*S*] (Eq. 6), if we know all activities $v_{i}^{*}[S]$ or weights $w_{ij}^{*}[S]$ for *S* ∈ [0,*P*]. For the model parameters we use, the equilibrium probability distributions obtained by this approximation closely resemble those from a full simulation of the dynamics (see Text S4).

### Classification of possible distributions

With Equation 2 we have a tool to calculate an approximation of the equilibrium distribution. With this equation, we now want to explore which qualitatively different shapes this distribution can take and how the interaction between neuron model, synaptic and structural plasticity influence it. For this, two distributions are considered to be qualitatively different, if they differ in number and arrangement of their local extrema (peaks and valleys).

As we will show, these extrema are already fully determined by Equation 2, which can, furthermore, be transformed such that the influences of the neuron model and structural plasticity can be mathematically treated independently.

To distinguish qualitatively different probability distributions *p*[*S*], we first identify the number and arrangement of their local extrema. For this, we treat *p*[*S*] as a function of *S*. Along this line, the logarithm of the ratios given in Equation 2

$$\Delta_{\ln p}[S]:=ln\left(\frac{p[S]}{p[S-1]}\right)=\underset{:=-p_{cf}}{\underset{︸}{\ln p_{build}+\ln\left(\frac{P-S+1}{S}\right)}}\underset{:=p_{d}}{\underset{︸}{-\ln p_{del}\left[w_{ij}^{*}\left[v_{i}[S],v_{j}\right]\right]}}$$ (3)

behaves like a discrete version of the derivative of this function. If Δ_ln*p*_[*S*] is positive, the function *p*[*S*] grows with *S*. If Δ_ln*p*_[*S*] is negative, *p*[*S*] will have smaller values for larger *S*. Therefore, as a necessary condition for extrema, hence peaks and valleys, in *p*[*S*], we simply have to determine the zero-crossings Δ_ln*p*_[*S*] = 0. Moreover, the sufficient condition for a peak would be a zero crossing from positive to negative values, and for valleys it is the other way around.

To see the influence of the plasticity models more clearly, we rewrite Equation 3 depending on postsynaptic activities $v_{i}^{*}$ in the following way: One can assume that the postsynaptic activity $v_{i}^{*}[S]$ grows (strictly monotonically) with the number of synapses. In this case we can invert this relationship to $S[v_{i}^{*}]$. Then, we can rewrite Δ_ln *p*_ as a function of the postsynaptic activity $v_{i}^{*}$. The zero-crossings of $\Delta_{\ln p}[v_{i}^{*}]$ are then given by the solutions of the equation

$$p_{cf}\left[v_{i}^{*}\right]=p_{d}\left[v_{i}^{*}\right]$$ (4)

$$\text{with }p_{cf}[v_{i}^{*}]\;:=\;-\ln p_{build}-\ln\left(\frac{P-S[v_{i}^{*}]+1}{S[v_{i}^{*}]}\right)$$

$$\text{and }p_{d}[v_{i}^{*}]\;:=\;-\ln p_{del}\left[w_{ij}^{*}\left[v_{i}^{*},v_{j}\right]\right].$$

Number and types of extrema of the equilibrium distribution of synapses in a connection is determined solely by the intersection topology (crossing points) of $p_{cf}[v_{i}^{*}]$ with $p_{d}[v_{i}^{*}]$, where fine details of these functions will not matter. This is an important notion as it allows us to analyse *types of* neural activation function interacting with *types of* plasticity rules asking whether or not their interaction will reproduce the biologically observed synaptic distributions. Hence, we can restrict the analysis to the different, possibly existing, generic cases how such crossing points could arise from different shapes of $p_{cf}[v_{i}^{*}]$ and $p_{d}[v_{i}^{*}]$.

**Figure 2:**
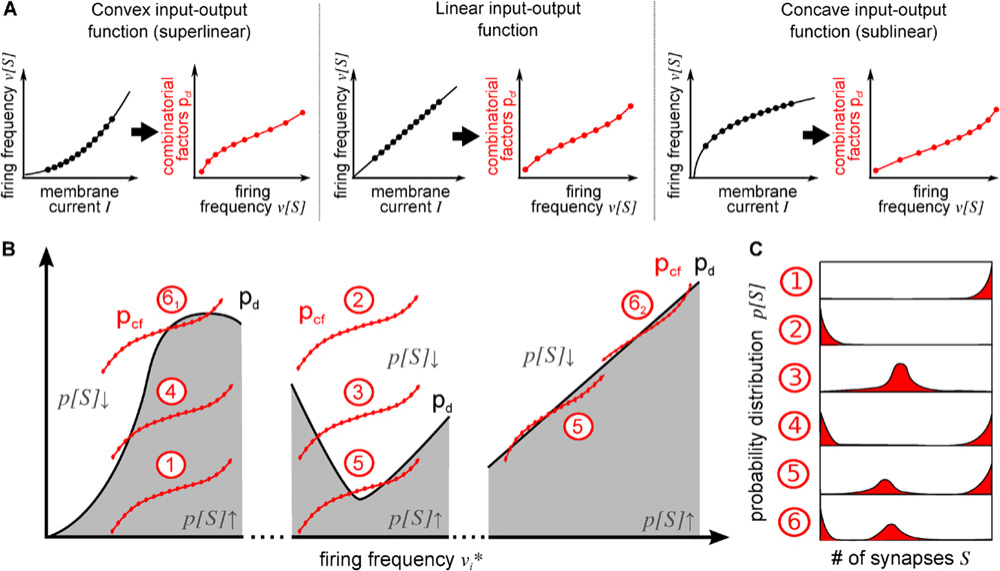
Possible distributions of the number of synapses of a single connection resulting from the interaction of synaptic and structural plasticity with different neuron models. *(A)* Three different curvatures of input-output functions *F* of the neuron (black) lead to different shapes (curvatures) of the combinatorial term *p*_*cf*_ (red, see Eq. 4). For fixed presynaptic activity and postsynaptic stimulation, the lines are calculated for continuous values of *S*, whereas the dots mark successive discrete values. *(B)* When the combinatorial influences *p*_*cf*_ (red, Eq. 4) are smaller than the logarithmic deletion probability *p*_*d*_ (black) for a certain value of *S* (grey shaded area), the long-term equilibrium probability for *S* synapses is higher than the probability for *S* − 1 synapses (see Eq. 2) and vice versa. Thus, intersections of both terms indicate peaks and valleys of the probability distribution *p*[*S*]. To cover all six possible intersection structures between *p*_*cf*_ and *p*_*d*_, we show example snippets for the *p*_*d*_ with a variety of curvatures and slopes. *(C)* The shape of the long-term equilibrium probability distributions (schematically) for the number of synapses of the plastic connection can be derived from the intersection structures in *(B)*: each intersection in *(B)* leads to a local extremum in the probability distribution in *(C)*. Furthermore there can be peaks at the boundaries. Note, experimental connectivity (Fig. 1A) corresponds to case six which has two intersections. As *p*_*cf*_ is monotonically growing, two intersections are only possible for growing *p*_*d*_-functions.

Note, in Equation 4 the influence of the deletion probability and synaptic plasticity is represented by *p*_*d*_. The term *p*_*cf*_ contains an offset term *p*_*build*_ and combinatorial factors, which are shaped by properties of the neuron model via $S[v_{i}^{*}]$: we find that that $p_{cf}[v_{i}^{*}]$ takes a characteristic S-shape (see Fig. 2A, middle), where curvature may vary following the input-output (neural activation) function (Fig. 2A, left and right). The function *p*_*d*_ changes slowly across variable input frequencies but could in principle take any shape. Still, under these constraints, there are only a few possible intersection topologies between $p_{cf}[v_{i}^{*}]$ with *p*_*d*_ existing, depicted in Fig. 2B. As long as we have slowly changing curvatures of the neural activation functions (Fig. 2A), which represents the biologically relevant situation, we will observe maximally two extrema of the synaptic distributions. We have sorted and numbered these six cases by the number of intersections or extrema in the probability distribution. The resulting qualitatively different probability distributions are sketched in Fig. 2C. It can be seen that the biologically observed case (Fig. 1A) is represented by case 6, but we will show in the following (see section “Connection between two neurons can show hysteresis”) that also some of the other cases play an important role for the dynamics of the system.

Although case 4 also shows two maxima at zero and at higher values, the second peak for number of synapses would only be at the maximum number of synapses P. Thus, the observed falling flank of the upper peak would have to stem from the distribution of potential synapses. As investigations of these distributions show a much shallower tail [40], case 4 will not be considered in the following.

**Table 1:** Distribution shapes for different numbers of extrema.

case	# roots Δ_ln *p*_	sgn Δ_ln *p*_	distribution description
1	0	+	one maximum at upper boundary (*P*)
2	0	-	one maximum at lower boundary (0)
3	1	+ -	maximum at position of sign-change (attractive fixed point)
4	1	- +	maxima at both boundaries, repulsive fixed point at sign-change
5	2	+ - +	maxima at first sign-change and upper boundary
6	2	- + -	maxima at lower boundary and second sign-change

### Constraints for biological realistic behaviour

As already mentioned, the experimental distributions of the number of synapses between two neurons [10, 36–39] are based on too small datasets to interpret them as a significant quantitative measurement. However, the shape of the probability distribution is quite similar for all datasets (Fig. 1A), such that we consider this qualitative shape to be a general property of biological neural networks (see Text S1). This common distribution shape exhibits a strong peak at *S* = 0 followed by a probability minimum for one or two synapses and a second peak between three to eight synapses. As this distribution shape corresponds to case six in Fig. 2C, we can identify the class of biological realistic plasticity rules and neuron models, which yield case six dynamics. Following the results from the previous section, this case necessarily needs two intersections between *p*_*d*_ and *p*_*cf*_ (i.e. necessary condition: two sign-changes of Δ_ln *p*_) at which the sign of Δ_ln *p*_ has to change from negative to positive and from positive to negative (sufficient condition). In the following, we will translate these conditions to properties of the neuron model and the plasticity rule. We will show that, as a necessary condition, the fixed synaptic weight must grow with the postsynaptic activity. Note, this describes a rather generic condition, which, however, is very often *not* fulfilled by standard learning rules. Nonetheless, we will show that a large class of realistic rules obeys this and especially if the system is embedded in a recurrent network.

**Necessary condition** In the first step, we determine under which conditions we can obtain two intersections between *p*_*d*_ and *p*_*cf*_: If we look at *p*_*cf*_, we find that it is always increasing with *S* and, thus, also with $v_{i}^{*}$. Therefore, any monotonously decreasing or constant $p_{d}[v_{i}^{*}]$ can intersect it maximally once. This would lead to maximally one sign-change of Δ_ln*p*_ and to the cases 1–4 (Fig. 2). Thus, to exhibit case six, $p_{d}[v_{i}^{*}]$ necessarily needs to have a positive slope. Following Equation 1, we obtain that $p_{d}\propto w_{ij}^{4/3}$ which is a monotonously increasing function of *w*_*ij*_. Thus, instead of investigating the slope of *p*_*d*_, we can also evaluate whether the derivative $dw_{ij}[v_{i}^{*},v_{j}]/dv_{i}^{*}$ is positive, i.e. whether the synaptic weight grows with the postsynaptic activity.

As the plastic connection can be part of a recurrent network, the postsynaptic activity can also influence the fixed weight by feeding back to the presynaptic activity. This dependency can be approximated by a Taylor series $v_{j}\approx r_{0}+r_{1}v_{i}+O[v_{i}^{2}]$. Note, here we take the unusual perspective of presynaptic activity as a function of the postsynaptic one. We restrict our following analysis to two representative systems: a feed-forward system (*r*_0_ = *const*,*r*_1_ = 0) and a linear-feedback system (*r*_0_ = 0, *r*_1_ = 1).

In these two systems we now evaluate, if the condition of weights growing with postsynaptic activity is met for the following commonly used rate-based learning rules: the Hebb rule and the fixed-threshold Bienenstock-Cooper-Munro (BCM) rule with hard boundaries on the weights, the Oja rule, the BCM rule with sliding threshold [18], and the Hebbian and the fixed-threshold BCM rule with weight-dependent synaptic scaling [25]. Although the time scales of synaptic scaling (minutes to days) and structural plasticity (days to months) slightly overlap, we still apply the Markov-system-approximation for the rules including synaptic scaling. This approximation is supported by the similarity of the probability distributions resulting from the (approximated) analysis and from the simulation of the full system dynamics (see Text S4).

Surprisingly, we find that among the investigated rules a positive slope of the $v_{i}^{*}-w_{ij}^{*}$-relation is only found by the Hebb rule with synaptic scaling in the linear feedback system or by the BCM rule with synaptic scaling in both systems, hence with and without feedback. Thus, our model predicts that, at least for Hebb-like synaptic plasticity, feedback plays a crucial role in generating the biological distribution of the number of synapses. This relates well to the finding that recurrent microcircuits are overproportionally abundant in cortical networks [34, 35]. When evaluating the $v_{i}^{*}-w_{ij}^{*}$-relation for a recently published calcium-based plasticity rule [24], we find that also this biologically more detailed rule shows a growing $v_{i}^{*}-w_{ij}^{*}$-relation. Although we only show a limited set of rules here, many other rules, as, e.g., Hebbian learning with soft bounds and weight decay [21], fulfil this necessary constraint (see Supporting Table S1 for more rules). In general, the analysis we present here can be used as a tool to judge whether a learning rule of interest has the potential to generate a certain connectivity.

**Figure 3:**
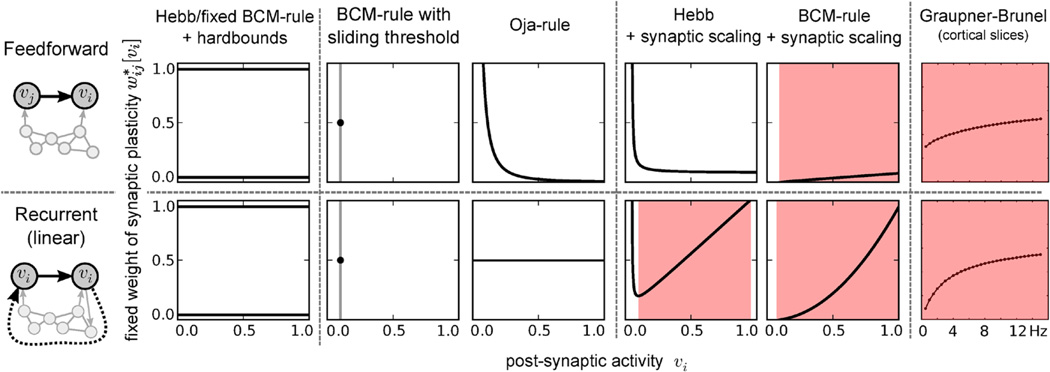
Most of the commonly used rate-based learning rules do not provide a positive correlation between weight and postsynaptic activity. The slope of the *p*_*d*_-term in Equation 2 is determined by the slope of the fixed weight depending on postsynaptic activity ($v_{i}^{*}-w_{ij}^{*}$ relation) resulting from the synaptic plasticity rule. Here we show the $v_{i}^{*}-w_{ij}^{*}$ relation of commonly used learning rules [18] for the simple feedforward system (top row) and for a linear approximation of a feedback system, where the presynaptic activity equals the postsynaptic activity (bottom row). For reproducing experimental data, *p*_*d*_ has to grow, i.e. the fixed weights have to increase with postsynaptic activity. This is fulfilled (red shaded area), for example, by the BCM-learning rule with synaptic scaling or a Hebb-like learning rule with synaptic scaling and feedback but also for more biological rules like the calcium-based plasticity rule from [24] (see Materials and Methods for parameters used to generate this figure).

**Sufficient condition** So far, we only set up a necessary condition for two extrema. In our second step, we ensure the right order of the extrema (minimum - maximum). For this, the average curvature of Δ_ln*p*_ must be negative (at least between the two zero crossings), i.e., the difference between the curvatures of *p*_*d*_ and *p*_*cf*_ must be negative. This could be achieved, either by a strong(er) negative curvature in *p*_*d*_ (compare case 6_1_ in Fig. 2B) or a strong(er) positive curvature in *p*_*cf*_ (compare case 6_2_ in Fig. 2B). As the common rate-based learning rules mostly do not lead to a strong negative curvature (compare Fig. 3), a positive curvature of *p*_*cf*_ seems to be more plausible. As shown above, this can be achieved by a neuron model which has an input-output-curve with negative curvature (concave) in the relevant interval of $v_{i}^{*}$ (resulting in case 6_2_). On the other hand, when assuming very low frequencies, where biological neurons typically have convex input-output-relations, it is more plausible that the relation between fixed weight and postsynaptic activity is sublinearly growing (i.e. negatively curved), which would result in case 6_1_. Although, the commonly used rate-based learning rules typically do not show this behaviour, there are synaptic plasticity rules which fulfil this constraint. For example the $v_{i}^{*}-w_{ij}^{*}$-relation of a calcium-based plasticity rule [24] fulfils the required behaviour (Fig. 3 rightmost panel). Thus, in either case, also the sufficient condition translates to a biologically reasonable constraint.

In summary, this analysis predicts that biological connectivity can be generated by a weight-modulated structural plasticity rule under biological reasonable constraints. We therefore conclude, that structural rewiring in cortex could be regulated by the synaptic weight or its morphological correlates.

### Experimental distributions can be explained in certain activity regions

We now want to verify that an example combination of neuron model and synaptic plasticity rule, which fulfils the above conditions, can account for experimental data - here the distribution of connections between cortical layer IV cells [36].

As input-output-curve for this example, we use the logistic function (1 + exp(−*x*))^−1^, which has the required negative curvature for positive inputs. Thus, one would expect that the experimental data can be explained for postsynaptic activities above 0.5. For the synaptic plasticity rule, we use a fixed-threshold BCM rule with weight-dependent synaptic scaling [25] in the feed-forward system (*r*_0_ = *const*, *r*_1_ = 0):

$$\frac{dw_{ij}}{dt}=\mu \left(v_{j}v_{i}(v_{i}-\theta )-\kappa^{-1}(v_{i}-v_{tss})w_{ij}^{2}\right)$$

where *θ* is the LTP / LTD threshold of the BCM rule, *κ* a parameter which determines the influence of a single presynaptic neuron on the input, and *v*_*tss*_ the characteristic activity of synaptic scaling [25]. The learning rate *μ* sets the time scale of the convergence of synaptic plasticity, but does not influence the equilibrium distribution as long as it is faster than the structural plasticity time scale.

**Figure 4:**
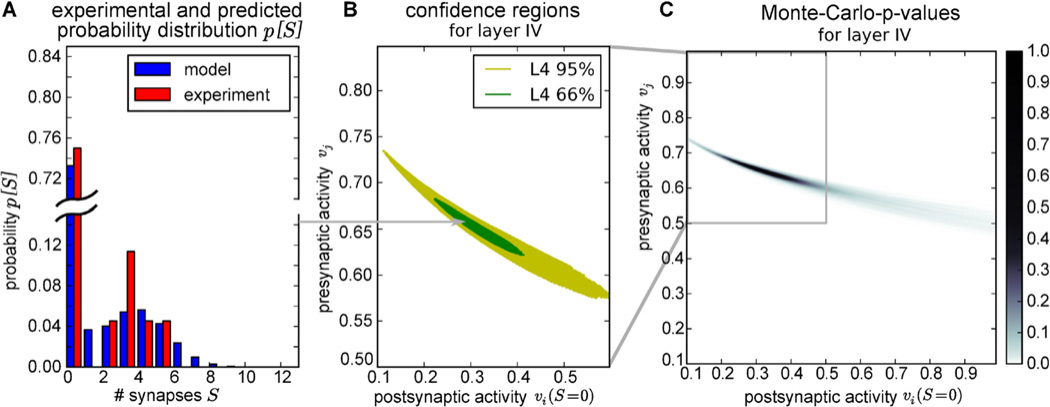
Model can account for experimental data for suitable pre- and postsynaptic activities. *(A)* The probability distribution of the number of synapses between two neurons from experiment ([36], red) is similar to the distribution resulting from the proposed model (blue) at *v*_*j*_ = 0.656 and *v*_*i*_(*S* = 0) = 0.2975. *(B)* The activity confidence regions, where error of the experimental outcome lies within the most probable 95% (yellow) or 66% (green) of the trials, when randomly sampling from model distribution, spans over a broad range of activities. (C) Colour code shows the Monte-Carlo p-values for the hypothesis that experimental data comes from model distribution. For comparability, postsynaptic influence *I* was transformed to the postsynaptic activity for *S* = 0 in all figures. (Parameters: BCM rule with synaptic scaling with *θ* = 0.08, *v*_*tss*_ = 0.1, *κ* = 9.0, structural plasticity: *P* = 12, ln *p*_*build*_ = −16, *a* = 2.0, *ρ* = 0.125)

For this model, we calculate the long-term equilibrium distributions *p*[*S*] for a broad range of presynaptic activities *v*_*j*_ and postsynaptic influences *I* (on a 446 x 357 grid). In Fig. 4A, one example equilibrium distribution for *v*_*j*_ = 0.656 and *v*_*i*_[*S* = 0] = 0.2975 is compared with the experimental distribution.

As the experimental distribution have so little statistics, standard statistical test fail to compare model and experiment. Therefore, we evaluated if the experimental distributions would be a probable outcome, when sampling from the model distribution randomly, by using a Monte-Carlo test (Fig. 4C, see Methods for details). Like in [40] we define 95% confidence regions as the activity regions, where the model distribution is statistical not significantly different from the experimental one (p-value > 5%), i.e. where the model can account for the experimental distribution (Fig. 4B). For a better comparability, the postsynaptic influence *I* is transformed to the resulting postsynaptic firing frequency $v_{i}^{*}[S=0]$ for zero synapses (without presynaptic influence). The resulting confidence region spans a broad range of presynaptic activities and postsynaptic stimulations. However, choosing one of the parameters restricts the other one quite strongly, which indicates, that data could stem from different activity levels, but depends strongly on the right combination between pre- and postsynaptic influences.

### Comparison of the activity regions for different cortical layers

**Figure 5:**
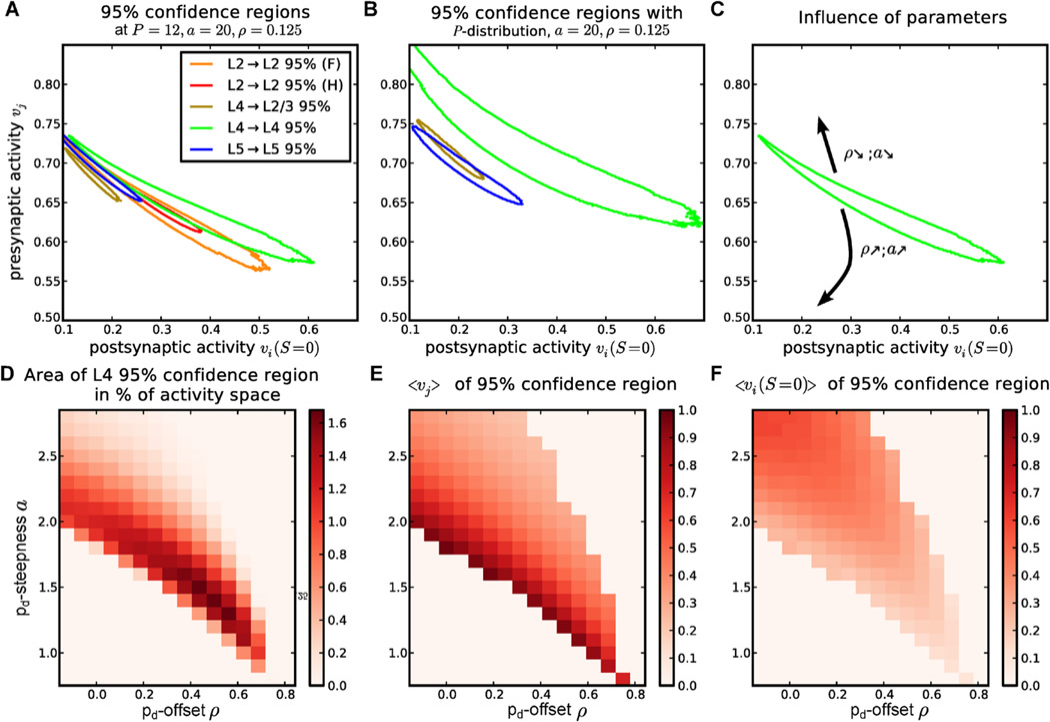
Different experimental data can be explained at different activity regions; effects are robust to underlying distributions or parameter changes. *(A)* The 95% confidence regions for experimental data from different cortical layers ([10, 36, 37], Fig. 1A) are located at different activities for different layers. Confidence regions for same experimental location (layer II) overlay. *(B)* The 95% confidence regions, which emerge from the same model parameters when using a distribution of potential synapses from [40], are qualitatively not different. *(C)*, Schematic drawing which summarises the influence of *a* and *ρ* on the location of the confidence region (see *(E)* and *(F)*). For the layer IV data, we evaluated how *(D)* the area in activity space, *(E)* the average presynaptic activity, and *(F)* the average postsynaptic influence of the activity confidence region changes for different structural plasticity parameters *a* and *ρ*. (Parameters BCM rule with synaptic scaling with *θ* = 0.08, *v*_*tss*_ = 0.1, *κ* = 9.0, structural plasticity: *P* = 12, ln *p*_*build*_ = −16, in *A-C*: *a* = 2.0, *ρ* = 0.125)

We now want to see if the set of plasticity parameters, which was used to account for the layer IV connections, would also be able to explain the distributions from other cortical layers and areas. Although the properties of neurons and synapses could be very different in different layers and areas, we analyse whether the activities can cause the differences in the experimental distributions. Therefore, we also calculate the confidence regions for connections between visual cortex layer V cells [10] and barrel cortex layer II/III cells [38, 39] as well as connections from barrel cortex layer IV to layer II [37] (Fig. 5A). We find that all distributions can be explained by the same model and parameters, but the activities where the model can account for the data differ for each dataset. However, these layer-specific activities are consistent with each other and relate well to biologically observed activities: When comparing the datasets which are taken from rats barrel cortex, we find that the connections from layer IV are on average explained by higher presynaptic activities than connections from layer II. Also, the postsynaptic activity seems to be higher for connections to layer IV. This corresponds to experimental observation of the spontaneous activities in the rat barrel cortex [45, 46] as well as model predictions of the responsiveness of different cortical layers [53]. In both cases, cortical layer IV exhibits higher activities than layer II. Furthermore, the two confidence regions for layer II/III experiments are perfectly overlapping, while - as expected - the dataset with more statistics leads to a smaller confidence region. This supports the hypothesis that activity can be the parameter which causes the differences of the experimental distributions. On the other hand, the confidence regions are also partly overlapping. This indicates, that different activities are not even necessary to explain the experiments. Either way, one parameter-set for the synapses can be used for all layers.

### Experimental data can be reproduced at reasonable frequencies

In the following, we obtain a quantitative estimate for the activities in different layers from a model which is more closely matched to biology. For this, we repeated the calculations of the activity confidence regions for layer II and layer IV intra-layer connections from somatosensory cortex with an adaptive exponential integrate-and-fire neuron [54] and the calcium-based spiking plasticity rule [24] described above. We first obtain the input-output-curve of the neuron when stimulated with a constant current. Then, we determine the fixed weight of the plasticity rule when stimulated with pre- and postsynaptic Poisson-spike trains as a function of their frequencies. Interpolation between the simulated values provides us with continuous functions for which we can apply the analysis described above.

**Figure 6:**
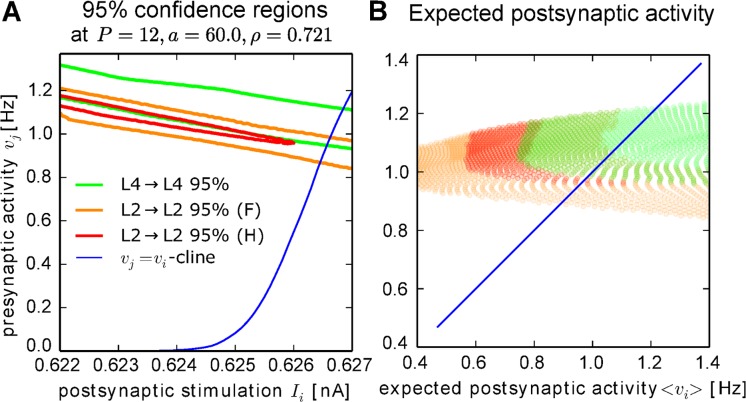
Quantitative estimates of the activities show biological reasonable ordering in a reasonable frequency regime. *(A)* Activity confidence regions for intra-layer connections in somatosensory cortex. To obtain quantitative results, we used an input-output-relation (blue line) from an adaptive exponential integrate-and-fire neuron [54] and a calcium-based plasticity rule [24] (see Methods). As in Fig. 5, the activities for layer IV connections are larger than those for layer II connections. The firing frequencies lie in a biological reasonable range. *(B)* The expectation value of the postsynaptic activity for stimulations within the confidence intervals confirm that experimental data can be reproduced with equal pre- and postsynaptic activity as expected for intra-layer connections.

As above, we find also for this biologically more reasonable system that the ordering of the activity confidence regions corresponds to the experimental measurements. Furthermore, we assumed that the pre- and postsynaptic activity must be equal for intra-layer connection. Along this line, we estimate the activities in different layers as the intersection between the *v*_*i*_ = *v*_*j*_-cline (blue line in Fig. 6) and the activity confidence region of that layer. For the *v*_*i*_ = *v*_*j*_-cline, we used both the baseline postsynaptic activity *v*_*i*_(*S* = 0) (Fig. 6A) and the expected postsynaptic activity calculated for the stationary distribution (Fig. 6B). Although the resulting frequencies do not match the experimental values exactly, our simple model, which uses the same input-output-relation for all neurons and neglects influences of recurrences or inhibition, predicts frequencies on the right order of magnitude [45] of about one Hertz.

### Influence of structural plasticity parameters

In this section, we demonstrate that the structural plasticity model does not require fine tuning to yield bimodal distributions and, thus, that they are a general feature of the proposed model. Along this line, we evaluate the influence of altered structural plasticity parameters on the size and position of the confidence region in activity space for the system used in Fig. 5A.

First, we investigate the influence of an altered number of potential synapses. For this, we use the estimated probability distributions of the number of potential synapses *p*[*P*] from [40] instead of the fixed number *P* = 12. Along this line, we calculate the equilibrium distributions for each *P* ∈ [1, 20] separately. Then, we sum those distributions weighted with the probabilities *p*[*P*]. Again, we test, if this summed distribution is statistically different from the experimental distribution and obtain 95% confidence regions (Fig. 5B). We find that the shape of the confidence regions resembles the one for *P* = 12 potential synapses, but is overall larger (see Fig. 5B), and that the confidence regions for the *P*-distributions are slightly shifted to higher presynaptic activities. This is not surprising, because the mean probability mass of the *P*-distribution lies below *P* = 12 and smaller *P* increase *p*_*cf*_ by decreasing the combinatorial factors. To maintain the same intersection structure for smaller *P*, larger weights and larger activities are required in *p*_*d*_.

Second, we vary the parameters *a* and *ρ* which determine height and width of the deletion probability *p*_*del*_. For each combination (*a*,*ρ*) we determined the area in the activity space as well as the averaged presynaptic activity and postsynaptic stimulation of the 95% confidence region for layer IV data [36] on a grid of 90x90 values (Fig. 5C-F).

It turns out that an increase in a *a* or *ρ* shifts the confidence regions to lower presynaptic activities and higher postsynaptic stimulations (Fig. 5E and F; schematically summarised in Fig. 5C ). Also, there is a corridor in the *a*−*ρ* space where the confidence regions reach a maximal area. The relation of *a* and *ρ* along that corridor follows a negative linear function (Fig. 5D). Only parameter sets far away from this maximal corridor eventually lead to a disappearance of the confidence region, and, in general, the system still shows the desired behaviour when the optimal parameters are varied by 10 – 20%.

For the neuron model and synaptic plasticity rule we use here, the largest area of the confidence regions is obtained when the presynaptic activity is larger than the postsynaptic baseline activity $v_{i}^{*}[S=0]$. Choosing approximately equal pre- and postsynaptic activities leads to smaller confidence regions in this system, but the model can still account for the data. A very large confidence region only means that the system behaviour is very similar over a large range of activities, which indicates that this behaviour is robust to changes in activity. On the contrary, a more confined confidence region means that a significant change of the distribution can be achieved by a smaller change in activity, which indicates that the connectivity can be controlled by changing the activities and stimulations more easily.

### Activity-dependent changes of the equilibrium distribution

The size of the confidence region, however, can only predict the sensitivity of the equilibrium distribution to activity changes. In the following, we answer the question how an altered activity changes the shape of the distribution. For this, we evaluate activity-dependent changes for the above example model which was already shown to exhibit biological realistic behaviour for a certain combination of pre- and postsynaptic influences. We now use this combination as a putative working point of the biological system, keep one of the influences at the value of this working point and calculate the equilibrium distributions when varying the other.

**Figure 7:**
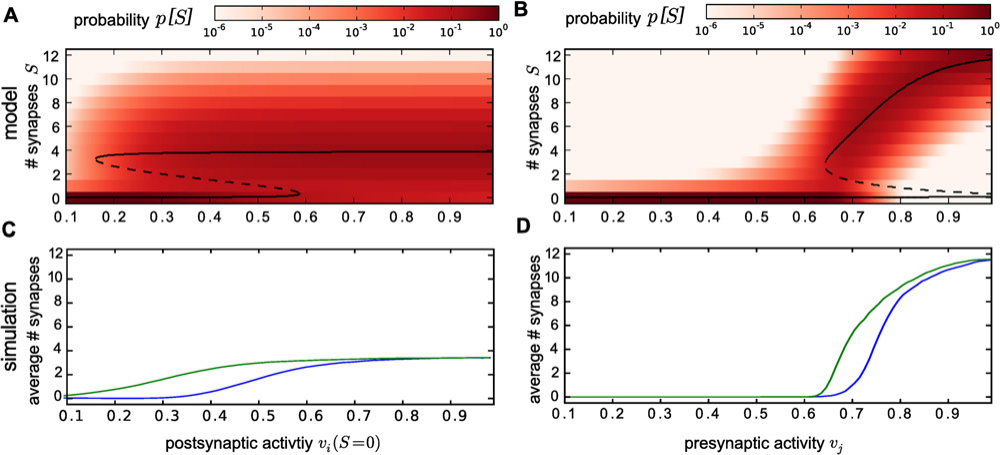
The BCM rule feedforward connection shows a hysteresis in pre- and postsynaptic stimulation. *(A)* The predicted probability distributions *p*[*S*] (in colour-code) for the system from Fig. 4A is strongly influenced by varying the postsynaptic stimulation. Black lines indicate the values of *S* (treated as continuous variable) for which synapse creation and deletion are equally probable. These points correspond to stable (continuous line) or unstable (dashed line) fixed points of the dynamical system following the net probability flow of our system and indicate the existence of local extrema in the long-term equilibrium probability distribution. Two bifurcations lead to an appearance and disappearance of a bistability, which indicates a possible hysteresis. *(B)* The same applies for varying the presynaptic activity, although the second bifurcation on the right hand side does not reveal for continuous *S*, but takes place in the discrete case as both sign changes happen between two consecutive states. *(C)* Simulation reveals the predicted hysteresis: postsynaptic stimulation was increased stepwise such that *v*_*i*_(*S* = 0) increased by steps of 0.01 until it reached 1.0 and then decreased again. For each stimulation, the average number of synapses was calculated separately for the in- and decreasing direction and later averaged over all stimulation cycles (see Methods). The blue curve depicts the average number of synapses in the increasing and the green curve the decreasing direction. *(D)* Altering presynaptic activity in the same way also yields a hysteresis loop. (Parameters: BCM rule with synaptic scaling with *μ* = 0.2, *θ* = 0.08; *υ*_*tss*_ = 0.1; *κ* = 9.0, structural plasticity *P* = 12; ln *p*_*build*_ = −16, *a* = 2.0, *ρ* = 0.125, in *A, C*: *v*_*j*_ = 0.656, in *B, D*: *v*_*i*_(*S* = 0) = 0.2975)

As predicted by the confidence regions, the shape of the distributions are altered strongly by these changes: If pre- or postsynaptic influences are weak, there is only one probability maximum at *S* = 0 (Fig. 7A for pre- and Fig. 7B for postsynaptic influence). For higher influences, the second peak emerges and for even stronger influences the probability mass shifts to the upper peak until the peak at *S* = 0 eventually vanishes.

In our example system, changing postsynaptic influence only appears to alter the height of the probability peaks, which are present at the working point, leaving their shape unchanged, whereas changing presynaptic activities also shift the position of the upper peak until it reaches the maximum number of synapses.

In order to understand this behaviour, we treat *S* as a continuous quantity and calculate the values of *S* where building and deleting a synapse are equally probable (Fig. 7A-B black lines). These points separate the states *S* (intervals for continuous *S*) where the system is expected to increase or decrease its number of synapses. If we treat the stochastic process like a dynamical system which would be following the net probability flow, these points correspond to attractive (solid line) and repulsive (dashed line) fixed points. If the net probability flow points towards one of the boundaries *S* = 0 or *S* = *P*, we also add this boundary as an attractive state.

We see that these fixed points correspond to the shape of the distributions: for both low pre- or postsynaptic stimulation there is only the attractive state at *S* = 0. For stronger stimulations a pair of repulsive and attractive states is generated (comparable to a saddle node bifurcation in dynamical systems). For high postsynaptic stimulation, a second bifurcation leads to the disappearance of the repulsive fixed-point and the attractive state at *S* = 0, leaving only the upper attractive state. For presynaptic activity the second bifurcation does not occur if *S* is a continuous variable. However, it effectively does take place when the two lower fixed points lie closer together than the discrete states of *S* can resolve.

As we showed above, a necessary condition for the existence of two fixed points is that the fixed-weights resulting from synaptic plasticity grow with increasing postsynaptic activities. Given this, synaptic plasticity maps an increase in the number of synapses onto larger synaptic weights, which finally results in a decrease of the deletion probability. At some point, synapse deletion becomes as probable as synapse creation. This point can be viewed as a threshold: All numbers of synapses below this threshold (below dashed line in Fig. 7A and B) yield systems which remove synapses more likely than they create them and converge to *S* = 0. For all higher numbers of synapses the system converges into the upper attractor. This bistability emerges from structural plasticity only due to the growing $v_{i}^{*}-w_{ij}^{*}$-relation. A bistability of the weights themselves, as observed, e.g., for the BCM rule, is not necessary and also not used here (e.g., our *v*_*i*_(*S* = 0) = 0.2975 is larger than the LTP/LTD-threshold *θ* = 0.08 of the BCM rule). However, a potential bistability of the weights could even strengthen the bimodality of the synapse-distributions and enhance the effects observed in this study.

Beside the bistability, we also observe regimes with one fixed point. In these regimes, synaptic plasticity does not only map changes in the number of synapses, but also the external pre- and postsynaptic stimulations onto the weights. For example, at very small stimulations, the weights are small and the deletion probability is much larger than the build-up probability. Therefore, the changes in the weights due to the number of synapses may not be sufficient to create a regime where synapse creation dominates such that there is no upper attractor.

These examples show that the activity-dependent change of the equilibrium distribution can only be understood from the interaction of synaptic and structural plasticity. Along that line, Equation 4 provides insight into the fixed points and bifurcations which govern the system dynamics.

### The connection between two neurons can show hysteresis

In dynamical systems theory, the bifurcation structure described above is associated with hysteresis [55]. A hysteresis, in turn, would mean, that temporary changes in activity leads to persistent changes in the system’s dynamic. This would be a very desirable feature of the system, as it indicates the possibility to store information in the connectivity. However, for a stochastic system a hysteresis does not necessarily emerge, because in contrast to a dynamical system which can remain in a certain state for infinite times without losing information, the stochastic dynamics in the long-term equilibrium yields a stationary distribution which is independent of the system’s history. But, if activities change faster than this equilibrium can be reached, the connection between two neurons could in principle exhibit a hysteresis. To test this, we simulate its behaviour while we repeatedly increase the presynaptic activity or postsynaptic influence stepwise until they are close saturation (*v* = 0.99) and then decrease them again to *v* = *v*_*tss*_. Thus, the system is on average exposed to an intermediate stimulation, where both attractors exist, but experiences both very high and very low stimulations during one stimulation cycle. As an indicator, whether the system behaves differently after low as compared to after high stimulation, we use the average number of synapses. We calculate this number for each step of such a stimulation cycle in the following way: the number of synapses is averaged over the time-interval with constant influences (separately for increasing and decreasing). Afterwards, the time-averages for each step are also averaged over all cycles.

Indeed, the average number of synapses shows a hysteresis loop when changing either influence (Fig. 7C and D). This means that the number of synapses can in fact depend on the system’s history. As already mentioned, this is only possible if the system does not reach its stationary probability distribution.

In the activity regime with two attractors, the probability that the Markov-Chain describing our structural plasticity process jumps from one basin of attraction to the other becomes very small. Thus, if the system is allowed to stay in the activity regime with two attractors for sufficiently small times in our simulation, we expect no transitions between the basins of attraction. Thereby, the two parts of the Markov-Chain become effectively unconnected (i.e. ergodicity breaking) and the system can be expected to stay in one basin of attraction. By de- or increasing the stimulation, one of the attractors vanishes and the whole Markov-Chain becomes effectively connected again with higher probabilities. Thus, the probability mass quickly shifts towards the remaining attractor. When the system is then brought back into the two-attractor regime, the Markov-chain separates again into two effectively unconnected parts and the system is very likely to stay in the attractor, it has been brought to.

However, the disconnection of the two parts is not due to a changing probability to jump to another state, as at least *p*_*build*_ is constant. Instead, the disconnection is a result of the low probability to be at the boundary of the basin of attraction, where creating (or deleting) one synapse already leads to a transition to the other basin of attraction. Thus, the probabilities *p*[*S*] of the number of synapses between the two peaks must be very low. As this is a feature of the experimental probability distributions, we expect all systems, which exhibit biological connectivity and a structural plasticity, which can be described similarly, to undergo this effective disconnection of the parts of the Markov-Chain and, thus, also to show a hysteresis. Furthermore, these low probabilities, to be at the boundary of the basin of attraction, can only emerge due to intermediate states between the two attractive fixed points. These states only exist for the multisynaptic system.

## Discussion

We presented a biological plausible, simple model for stochastic structural plasticity in adult networks and analysed its interaction with different synaptic plasticity rules and neuron models. Remarkably, we find that biological connectivity, i.e. the probability distribution of the number of synapses from one neuron to another, can only be explained when this structural plasticity interacts with synaptic plasticity rules leading to increasing synaptic weights along with stronger postsynaptic activities, which corresponds to the basic idea of Hebbian plasticity [5]. Additionally, the firing frequency of the neuron likely grows sublinearly with the input current. We further show that a model, which fulfils these constraints, can account for experimental datasets from different cortical layers at different activities, which are ordered in the same way as experimentally observed activities in those layers. Furthermore, although this is not explicitly implemented in our model, the connectivity can be controlled via pre- and postsynaptic stimulation. Along this line, we demonstrate that the number of synapses may exhibit a hysteresis when altering the pre- or postsynaptic stimulation. The hysteresis emerges from a very small probability that the system is close to the boundary of its actual basin of attraction. This can be assumed for all systems which exhibit the experimentally observed connectivity in long-term equilibrium.

### Faster synaptic plasticity mechanisms and fluctuations

In this work, we use rate-based neurons and rate-based synaptic plasticity rules. However, there exist synaptic plasticity mechanisms on faster time scales, as, for instance, spike-timing-dependent plasticity (STDP, [56]). On the time scale of our model, the effects of such plasticity mechanisms would appear as fluctuations of the weight or fluctuation of the volume of the corresponding dendritic spine respectively.

Accordingly, it has also been shown that the volume fluctuations partly depend on NMDA-receptor activation [13], which is associated with STDP. However, blocking of the NMDA-receptors shows that there are also other fluctuation sources.

As a consequence of such fluctuation, the weights or volumes are rather broadly distributed. Still, for each weight or volume in the distribution there is a certain probability for deletion in a certain time interval, as the fluctuations could have driven it beyond some deletion threshold. As both, the individual deletion probability and the distribution are unknown, we assume that one can directly calculate the expected deletion probability for the whole weight (volume) distribution from the characteristic weight *w*_*ij*_ emerging from synaptic plasticity via equation 1. Thus, the fluctuations can be viewed as the underlying reason for the stochastic deletion in this model and faster plasticity mechanisms should be already implicitly included. Along this line, the parameter *a* would correlate with the width of the resulting weight distribution and, so, with the strength of the fluctuations. As already described, volume or weight fluctuations are the basis of the synapse deletion probability in our model, such that we hypothesise that it already covers the influence of faster plasticity mechanisms.

The above argumentation relies on the assumption that spike-timing-dependent plasticity on average leads to undirected changes of the weight, which can be modelled as fluctuations. However, persistent strong temporal correlation between neural activities could alter the resulting synaptic weights strongly. In the case when the weights only depend on correlation of the firing between pre- and postsynaptic neuron, we could use this correlation instead of the postsynaptic firing in our analysis. Then, to fulfil the necessary condition for case six, the weight would have to grow with this correlation. Moreover, it is also reasonable that the correlation saturates at some point and, thus, also the sufficient condition can be fulfilled. Therefore, even when synaptic weights depend on correlations between the neuronal firing, the essential statements of our analysis remain true.

Yet, firing correlations and rates can also jointly determine the synaptic weight, together with many other factors like conduction delays, sub-threshold potentials [57] or molecular concentrations [24]. In that case, the interaction between the plasticity mechanisms would yield even more complex dynamics and our analysis has to be extended by these mechanisms.

### Potential synapses

In the presented model, we assume that the number of potential synapses between two neurons is constant. For adult networks, this is realistic, because the number of potential synapses is derived from the morphology of axons and dendrites, which has been observed to be quite stable in adult networks [12, 58]. However, there is evidence for large-scale morphological changes of axons and dendrites during development or after major injuries [1, 59]. Moreover, changes of the morphology depend on calcium concentration [60–62], which can be seen as a low pass filtered version of the neural activity. Thus, in order to account for early development or recovery from major injuries, our model would have to be extended by a dynamic number of potential synapses, which depends on the neuronal activity (see, e.g., [28]). However, for healthy adult networks this seems not to be necessary.

### Activity-dependent synapse formation or removal

Although experiments show an experience-dependent rate of spine formation [42, 49, 52, 63] or removal, the presented model assumes activity-independent synapse formation or removal probabilities. We do so because these effects might as well be a result of experience-dependent synaptic plasticity of the weights which leads to stabilisation or destabilisation of the corresponding dendritic spines [64, 65] without any direct activity dependence in the building or removal probability. However, we want to shortly discuss if and how an explicit activity-dependence of the probabilities would qualitatively change the presented results. For this, one has to consider that the equilibrium distributions of the number of synapses between two neurons are dominantly determined by the *ratio* between building and removal probability. Thus, if both formation and removal probability change similarly, their ratio, and, thus, the equilibrium probability distributions of the number of synapses, will not change and our results remain valid. Only the dynamics of the system would become faster or slower.

Otherwise, when the activity-dependent changes can be written as factors in *p*_*del*_ and *p*_*build*_, only the ratio of those changes will influence the equilibrium probability distribution, while the absolute values will only influence the speed of convergence. Thus, when only considering the equilibrium probabilities, the whole activity-dependence can be modelled by a activity dependence of one of the probabilities: e.g., a building probability which increases with activity (compare [66]) corresponds to a deletion probability which decreases with activity. Both cases can be modelled by a (postsynaptic) activity-dependent term with a negative slope in *p*_*d*_. As a consequence of that, the $v_{i}^{*}-w_{ij}^{*}$-relation would need an even stronger positive slope to fulfil the necessary condition.

Only when the building probability decreases or the removal probability increases with activity (homoeostasis) the dynamic of the system would change qualitatively and would not yield the same constraints as we find in our analysis.

### Information storage

The topology of neuronal networks has always been proposed to provide information storage capacity. Accordingly, the emergence of a hysteresis implies that the dynamic of a single connection is influenced by its past and, thus, also stores information about it. Systems with similar attractor structures (hysteresis) have been shown to store information, e.g., in computer-hard-drives but also in biological [67] and neuronal systems [68, 69].

It has already been shown that an autonomous network with homoeostatic structural plasticity follows a hysteresis during connectivity build-up in a self-organized way [26]. In contrast, the hysteresis loop in the current study is controlled by external stimulations.

In previous studies, the storable information of a single synapse is deduced from the number of possible topologies of a network, given by the number of possibilities to select a certain number of synapses from the pool of potential synapses [70, 71]. This relies on the assumption, that two different selections of the same number of synapses can be distinguished, which is only possible when considering the morphology of the neural system. However, the behaviourally relevant output of neural networks is rather determined by neuronal and synaptic dynamics than by morphology itself. Thus, we suggest that two topologies must be distinguishable from the network dynamics in order to represent two different states. In our model, by construction, all possible choices of the same number of synapses from one presynaptic neuron yield the same postsynaptic dynamic. Thus, they cannot be distinguished and code the same state. This leads to a decrease in the information capacity per synapse, which could be prevented by extending our model by morphology or other ways to distinguish multiple synapses (delays, shapes of postsynaptic potentials, etc.).

A further decrease in information capacity per synapse can be expected from fluctuations of the number of synapses. For example, on the time scale of the shown hysteresis, these fluctuations effectively allow us to determine only the basin of attraction the system is in. Thus, the sum of the information capacity of all synapses on one connection is maximally one bit.

The advantage of a multiple-synapse connection for information storage as compared to single-synapse systems reveals itself in the duration of learning and forgetting: The system is fluctuating around the attractive states and only transits to the other basin of attraction with a very low probability. This implies that the transition- or forgetting-time of the system exceeds the time, which is needed to build or remove a single synapse.

It was shown that in a structurally similar class of models - the so-called cascade models [72, 73] - the lifetime of memories is enhanced. In these models, connections can be in a weak or a strong state (comparable to the two basins of attraction in our model) consisting of several sub-states (number of synapses). Two plasticity mechanisms, synaptic plasticity and metaplasticity, are modelled by stochastic transitions between states and sub-states respectively. In our model, both transitions would be changes in the number of synapses, which either end up in the same or in the other attractor. Although, in our model, the sub-states are not exactly arranged as a cascade, the dynamics of the single connection is determined by the interaction of multiple exponential processes with widely ranging time scales (see Text S6). Due to the structural similarity, we would expect in our system similar power-law forgetting of stored memories as in the cascade model, which relates well to forgetting measured in humans [74–77].

Besides the functional form of the forgetting curve, another important problem has to be solved to model learning by a neuronal system: the system must always be plastic enough to form new memories, but also keep traces of old memories during acquiring new ones. This problem has been termed the plasticity-stability dilemma [78]. In the context of synaptic plasticity, neuronal systems with bistable synapses, which only switch their state due to extraordinary (high or low) activities [79], have been proposed to solve this problem [80]. In the context of structural changes, the results from above as well as previous work [40, 43] suggest that bistable dynamics also govern the number of synapses between two neurons. Our results now demonstrate that collective dynamics of synapses can store information about the system’s history and that, comparable to synaptic plasticity case, the number of synapses can be shifted to either basin of attraction due to high or low activities. Thus, we expect that the here proposed interaction of synaptic plasticity and structural plasticity can be used similarly to tackle the plasticity-stability dilemma for memories stored in the network structure.

## Materials and Methods

### Model

Each connection from neuron *j* to *i* in our model has a certain number of potential synapses *P*_*ij*_. At each time step of the simulation and each of those locations, the probability to create a functional synapse is *p*_*build*_ = *const*. The number of realised synapses is denoted by *S*_*ij*_ and each of these synapses has a weight *w*_*ij*,*k*_ with *k* ∈ {1, …, *S*_*ij*_}. The time development of those weights is described by a synaptic plasticity rule, a differential equation for the weight *w*_*ij*,*k*_, which only depends on local quantities accessible by the synapse (pre- and postsynaptic activities (*v*_*j*_,*v*_*i*_) and the weight itself) and is required to have a stable fixed point $w_{ij}^{*}[v_{i},v_{j}]$. Every realised synapse can be deleted with a weight-dependent probability

$$p_{del}[w_{ij,k}]=p_{build}^{\rho }\exp\left(-a^{2}w_{ij,k}^{4/3}\right).$$

Neuronal activities are determined by a nonlinear function *F* (the input-output curve) of the inflowing currents:

$$v_{i}=F\left[\sum_{j\neq i}\sum_{k=1}^{S_{ij}}w_{ij,k}v_{j}+I_{i}\right]$$

where *I*_*i*_ denotes neuron specific influences from outside the modelled network, e.g., inhibitory input or thalamic afferents. For the function *F* we use the sigmoidal function *F*[*x*] = (1 + exp(−*x*))^−1^ if not stated differently.

### Equilibrium distribution

Synaptic plasticity is fast compared to structural changes [48]. Thus, we assume that the weight has converged to its fixed point $w_{ij}^{*}$ before a structural change takes place. As the fixed weight and the corresponding fixed postsynaptic activity $v_{i}^{*}$ can be determined only from the actual number of synapses *S*_*ij*_, the deletion probability also only depends on *S*_*ij*_. Thus, if we interpret the number of synapses as states of the system, the transition probability to any other state only depends on the actual state. Therefore, similar as in [43], the system can be treated as a Markov process. The transition probabilities from *l* to *k* synapses are given by

$$M_{kl}=\{\begin{array}{ll} \sum_{x=0}^{\min\{l,P-k\}}\underset{\text{remove x synapses}}{\underset{︸}{\left(\begin{array}{c} l\\ x \end{array}\right)p_{del,l}^{x}\cdot {\left(1-p_{del,l}\right)}^{l-x}}}\underset{form\;k\;-\;l\;+\;x\;synapses}{\underset{︸}{\left(\begin{array}{c} P-l\\ k+x-l \end{array}\right){\left(1-p_{build}\right)}^{P-k-x}\cdot p_{build}^{k+x-l}}} & \text{if }k>l\\ \sum_{x=0}^{\min\{k,P-l\}}\underset{remove\;l\;-\;k\;+\;x\;synapses}{\underset{︸}{\left(\begin{array}{c} l\\ l-k+x \end{array}\right)p_{del,l}^{l-k+x}\cdot {\left(1-p_{del,l}\right)}^{k-x}}}\underset{form\;x\;synapses}{\underset{︸}{\left(\begin{array}{c} P-l\\ x \end{array}\right){\left(1-p_{build}\right)}^{P-l-x}\cdot p_{build}^{x}}} & \text{if }k\leq l. \end{array}$$ (5)

where $p_{dell}=p_{del}\left(w_{ij}^{*}[v_{i}[S=l],v_{j}]\right)$. As this is a strictly positive stochastic matrix, the Frobenius-Perron-theorem guarantees the existence of a stationary distribution, which can be calculated as the eigenvector for eigenvalue 1. This calculation may be numerically difficult as the entries of the matrix are distributed over many orders of magnitude (e.g., $p_{build}^{0}\ldots p_{build}^{P_{ij}}$).

However, to estimate the stationary distribution analytically one can use the first step approximation [43]. In this approximation, we allow the system only to increase or decrease its number of synapses by one during one time step. Thus, the states of the Markov-process are connected as a sequence. Once the system has reached its stationary state, the system is in detailed balance, i.e. the probability flow between two neighbouring states *S* − 1 and *S* cancel out, such that the probability of either state remains constant:

p[(S−1)→S]=p[S→(S−1)] (detailed balance)

$$\text{ with }p[(S-1)\to S]\;=\underset{\text{state probability}}{\underset{︸}{p[S-1]}}\;\cdot \;\underset{\text{transition probability}}{\underset{︸}{(P-S+1)\cdot p_{build}}}\;$$

$$p[S\to (S-1)]\;=\underset{\text{state probability}}{\underset{︸}{p[S]}}\;\cdot \;\underset{\text{transition probability}}{\underset{︸}{S\cdot p_{del}\left[w_{ij}^{*}\left[v_{i}[S],v_{j}\right]\right]}}\;.$$

From this we calculate the ratio between the probabilities of two neighbouring states in the stationary probability distribution

$$\frac{p[S]}{p[S-1]}=\frac{\left(P-S+1\right)}{S}\frac{p_{build}}{p_{del}\left[w_{ij}^{*}\left[v_{i}[S],v_{j}\right]\right]}$$

only by knowing the fixed weights $w_{ij}^{*}\left[v_{i}[S],v_{j}\right]$ for each number of synapses. The state probabilities can now be recursively calculated from *p*[0]

$$p[S]=p[0]\cdot \left(\begin{array}{c} P\\ S \end{array}\right)p_{build}^{S}\prod_{\hat{S}=1}^{S}p_{del,\hat{S}}^{-1}\text{ with }p_{del,S}:=p_{del}\left[w_{ij}^{*}\left[v_{i}[S],v_{j}\right]\right].$$ (6)

Finally, *p*[0] can be obtained from normalization

$$p[0]={\left(\sum_{S=0}^{P}\left(\begin{array}{c} P\\ S \end{array}\right)p_{build}^{S}\prod_{\hat{S}=0}^{S}p_{del,\hat{S}}^{-1}\right)}^{-1}.$$ (7)

### Investigated synaptic plasticity rules

The investigated synaptic plasticity rules [18–20, 25] are summarised in Table 2.

**Table 2:** Rate-based synaptic plasticity rules and their $v_{i}^{*}-w_{ij}^{*}$-dependencies.

**rule**	**differential equation** ${\dot{w}}_{ij}$	**feedforward** $w_{ij}^{*}$	**feedback** $w_{ij}^{*}$
Hebb with hard boundaries	${\dot{w}}_{ij}=\mu v_{j}v_{i}$ with *w_ij_* ∈ [*w_min_*, *w_max_*]	*w*_*max*_	*w*_*max*_
Bienenstock-Cooper-Munro rule … with fixed-*θ* and hard boundaries … with sliding threshold *θ* = *θ*[*t*]	${\dot{w}}_{ij}=\mu \cdot v_{j}v_{i}(v_{i}-\theta )$ with *w*_*ij*_ ∈ [0,*w*_*max*_] fixed point: $\theta \to \tilde{\theta }\cdot v_{i}^{*}{}^{2}$	*w*_*min*_; *w*_*max*_ $\frac{F^{-1}[{\tilde{\theta }}^{-1}]-I_{i}}{v_{j}}$	*w*_*min*_; *w*_*max*_ $\frac{F^{-1}[{\tilde{\theta }}^{-1}]-I_{i}}{{\tilde{\theta }}^{-1}}$
Oja rule	${\dot{w}}_{ij}=\mu (v_{j}v_{i}-w_{ij}v_{i}^{2})$	$\frac{v_{j}}{v_{i}}$	1
Hebb rule with weight-dependent scaling	${\dot{w}}_{ij}=\mu v_{j}v_{i}-\mu \kappa^{-1}(v_{i}-v_{tss})w_{ij}^{2}$	$\sqrt{\frac{\kappa v_{i}v_{j}}{v_{i}-v_{tss}}}$	$\sqrt{\frac{\kappa v_{i}^{2}}{v_{i}-v_{tss}}}$
fixed-*θ* BCM rule with weight-dependent scaling	${\dot{w}}_{ij}=\mu v_{j}v_{i}(v_{i}-\theta )-\mu \kappa^{-1}(v_{i}-v_{tss})w_{ij}^{2}$	$\sqrt{\frac{\kappa v_{j}v_{i}(v_{i}-\theta )}{v_{i}-v_{tss}}}$	$\sqrt{\frac{\kappa v_{i}^{2}(v_{i}-\theta )}{v_{i}-v_{tss}}}$

#### Parameters and their values used for Figure 3

Weight boundaries *w*_*min*_ = 0.04,*w*_*max*_ = 0.95; learning rate *μ* arbitrary; presynaptic activity *v*_*j*_ = 0.08; synaptic scaling characteristic activity *v*_*tss*_ = 0.05; synaptic scaling velocity parameter *κ* = 1; BCM threshold *θ* = 0.1; fixed weight for BCM with sliding threshold 0.5 (omitting any assumptions about the inverse input-output-curve *F* ^−1^ and inverse target activity $\tilde{\theta }$ of BCM ). The last panel was obtained from simulating the spike-timing-dependent plasticity rule from [24] with the therein provided parameters for cortical neurons (see below).

### Simulation of the calcium-based plasticity rule

As an example of a realistic spiking plasticity rule, we simulated the calcium-based plasticity rule proposed in [24]:

$$\tau_{w}\frac{dw(t)}{dt}=\gamma_{p}(1-w(t))\Theta [c(t)-\theta_{p}]-\gamma_{d}w(t)\Theta [c(t)-\theta_{d}]\;$$

$$\frac{dc(t)}{dt}\;=-c(t)/\tau_{c}+\sum_{k}C_{\text{pre}}\delta (t-t_{pre,k})+\sum_{l}C_{\text{post}}\delta (t-t_{post,l})$$

where Θ denotes the Heavyside-stepfunction and *t*_*pre*,*k*_ and *t*_*post*,*l*_ are the times of the *k*th presynaptic spike and the *l*th postsynaptic spike. We neglected the bistable potential term, as such a variation can be assumed to have no effect on the ability of this rule to account for various plasticity experiments [24]. However, this allows us to integrate the differential equations analytically between two occurring spikes (see Text S7). At low rates, this integration method allows simulation until weights have converged.

To obtain the fixed weight for a certain combination of pre- and postsynaptic rates, 500 connections were simulated simultaneously with stimulation from independent Poisson-inputs with these rates. After fixed time intervals, which covered at least 200 spikes from each site, the mean and standard deviations of the weights in the ensemble were evaluated. The simulation was stopped when the mean fluctuated around a stationary value and these fluctuations were smaller than the target accuracy of 0.005. For both, Fig. 3 and 6, the parameters for cortical slices were used [24]: *τ*_*w*_ = 346.361 s, *θ*_*p*_ = 1.3, *γ*_*p*_ = 725.085, *θ*_*d*_ = 1.0, *γ*_*d*_ = 331.909 *τ*_*c*_ = 22.7 ms, *C*_*pre*_ = 0.5617, *C*_*post*_ = 1.2396. For the feedforward system in Fig. 3, we used a presynaptic activity of *v*_*j*_ = 8 Hz. The error bars represent one standard deviation.

### Monte Carlo p-values

As the experimental datasets [10, 36–39] only contain little statistics and several numbers of synapses which were not observed, standard statistical tests are not suitable for our study. Instead, we use the following method to generate p-values to test if the experimental data can result from a model distribution *p*[*S*]: we sample from this distribution *N*_*exp*_ times, where *N*_*exp*_ represents the number of neuron pairs investigated in the experiments (this number sometimes had to be estimated by dividing the number of connected pairs by the connection probability in that experiment). The sampled data is sorted into a relative frequency histogram *p*_*sample*_ and compared to the model distribution *p*[*S*] by determining the squared error

$$SE:=\sum_{S=0}^{P}{\left(p[S]-p_{sample}[S]\right)}^{2}.$$

This process is repeated for *N*_*MC*_ times (typically *N*_*MC*_ = 1000) to obtain an estimate for the probability distribution of the error *SE* which results from randomly sampling from *p*[*S*]. Finally, we evaluate the squared error *SE* for the experimental distribution *p*_*exp*_[*S*] and determine the p-value as the probability to obtain a larger squared error from random sampling.

### Confidence regions of the adaptive exponential integrate-and-fire neuron

We simulated the adaptive integrate-and-fire neuron given in [54] stimulated by noisy current *I* (*σ*_*noise*_ = 0.05⋅*I*) with a forward Euler algorithm. The average spiking rate as a function of the input current was determined over intervals of 2500 s with currents increasing by steps of 0.5 pA in an interval from 600 pA to 1450 pA. The weights resulting from the calcium-based plasticity rule were simulated as described above and scaled with *w*_0_ = 0,035 nA Hz^−1^. Both simulated curves were then transformed to continuous functions by interpolating between the simulated values. For those functions, the fixed postsynaptic activities and weights were solved and used in the further analysis as described for Figs. 4 and 5.

### Simulations

Simulations were carried out in *C*. For each time step of the simulation, the following calculations were performed for all neurons: (1) calculate new firing rates from the standard sigmoidal input-output curve; (2) calculate weights from the learning rule by using a classical Runge-Kutta algorithm (4th order) for integration; (3) calculate deletion probabilities and delete synapses; (4) create synapses at vacant potential synapses. New weights were initialized with $w_{0}=0.05\sqrt{\kappa }{\left(1-v_{tss}\right)}^{-1/2}$.

For the fixed-threshold BCM rule with weight-dependent scaling

$$\frac{dw_{ij}}{dt}=\mu \left(v_{j}v_{i}(v_{i}-\theta )-\kappa^{-1}(v_{i}-v_{tss})w_{ij}^{2}\right)$$

the following parameters were used in all simulations: *μ* = 0.2, *κ* = 9, *θ* = 0.08, and *v*_*tss*_ = 0.1.

The synapse creation probability was set to *p*_*build*_ = exp(−16.0) and the removal probability was determined using *ρ* = 0.125 and *a* = 2.0.

To obtain the hysteresis curves, the stimulations *I*_{*i*,*j*}_ were repeatedly altered in a way that either the presynaptic activity or the postsynaptic activity at *S* = 0 synapses increased in steps of 0.01 until they reached 1.0 and then decreased again until they reached 0.05 (= 1 stimulation cycle). Each of those stimulations was applied for an interval of 6 ⋅ 10^5^ time steps for the postsynaptic and 6 ⋅ 10^6^ steps for presynaptic hysteresis curve. For each of those stimulation intervals, the average number of synapses of the stimulation-interval was saved. After simulation, these time averages were averaged over all stimulation cycles (2612 for postsynaptic and 1141 for presynaptic hysteresis).

## Supporting Information

Supporting Text S1 Error estimation for experimental datasets. In this text we estimate confidence intervals for the experimentally obtained probability distributions for the number of synapses between two neurons and evaluate error measures from previous work [43] which would result from these intervals.
(PDF)

Supporting Text S2 Derivation of the fixed-points of the different plasticity rules. (PDF)

Supporting Text S3 Input-Output-relations of different neuron models. We simulated the Input-Output relations of different neuron models to demonstrate that a concave Input-Output-relations is a common feature.
(PDF)

Supporting Text S4 Comparison of predicted distribution with simulations. For layer IV, the probability distributions from the experiment and the analysis with approximations are compared to a distribution obtained from a full simulation of the model dynamics.
(PDF)

Supporting Text S5 Sensitivity to the power of *w*_*ij*_. We show that the demonstrated effects do not strongly depend on the $w_{ij}^{4/3}$-dependency in the deletion probability by repeating the analysis from Fig. 4 to 5 for a $w_{ij}^{2}$-dependency.
(PDF)

Supporting Text S6 Relation to cascade model. In this text the similarities and differences to the cascade model are discussed in greater detail.
(PDF)

Supporting Text S7 Integration of the Graupner-Brunel plasticity rule. This text describes how the calcium based plasticity rule [24] is analytically integrated for the simulations.
(PDF)

Supporting Table S1 Learning rules and conditions. For a broad variety of learning rules this table indicates whether the fixed weights fulfil the necessary or the sufficient condition.
(PDF)
