# Supplementary material for: The Formation of Multi-synaptic Connections by the Interaction of Synaptic and Structural Plasticity and Their Functional Consequences
Source: PLoS Comput Biol. 2015 Jan 15;11(1):e1004031. doi: 10.1371/journal.pcbi.1004031 (PMC4295841; doi:10.1371/journal.pcbi.1004031)
Supplement: Supporting Text S1 — In this text we estimate confidence intervals for the experimentally obtained probability distributions for the number of synapses between two neurons and evaluate error measures from previous work [43] which would result from these intervals. (PDF) [file pcbi.1004031.s001.pdf]

Supporting Information for

The formation of multi-synaptic connections by the interaction of synaptic and structural plasticity and their functional consequences

Michael Fauth*, Florentin Wörgötter, Christian Tetzlaff
∗ E-mail: mfauth@gwdg.de

Error estimation for experimental datasets

In order to determine how reliable the experimental data is and, thus, how closely the experimental distribution must be matched by a model, we estimate the errors which emerge from the small sample size of the experimental data. For this, we use two different methods to determine the confidence intervals for the experimental distributions and the residual value R as defined in [43] for comparability:

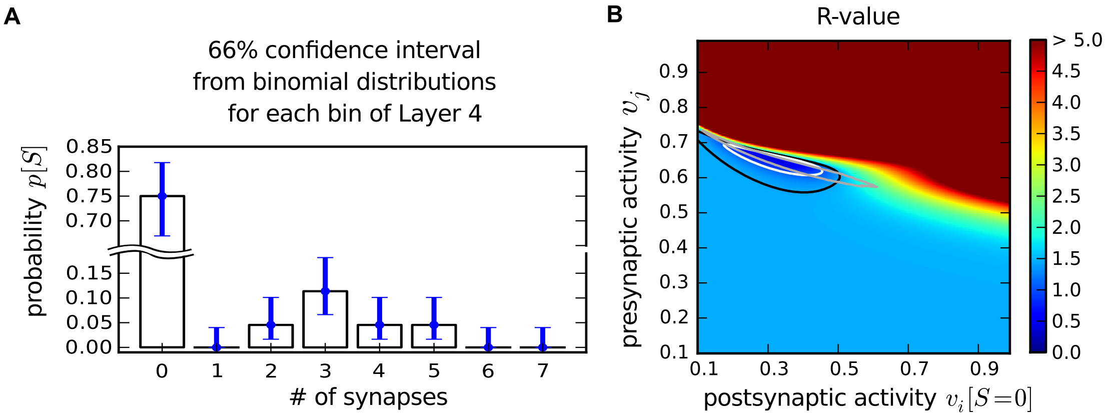

Supporting Figure S1. Estimated errors of the experimental distribution and activity regions with smaller errors. *(A)* Estimated confidence intervals for the probabilities of each number of synapses determined from Beta-quantile method. *(B)* R-values as defined in [43] for the same parameters as in Figure 4. *Black contour:* Isocline of the maximum possible R-value with the errors depicted in A. *White contour:* Isocline of the 95% confidence interval of the R value obtained from bootstrapping. The *grey contour* is the 95% confidence interval of the Monte-Carlo test from Figure 4. It can be seen that regions with low p-values also mostly have smaller R-values than necessary to account for the data.

Estimating the 95% confidence interval of R from bootstrapping: In this method, the experimentally observed probability distribution $p_{exp}[S]$ is used as an estimator of the real probability distribution. To estimate the distribution of the residuals R, as defined in [43], we sample from the experimental distribution as often as there are data-points in the experimental datasets (N_{exp}) and calculate a probability distribution $p_{sample}[S]$ from the relative frequencies. Then, we calculate the R-value between the experimental distribution p_{exp} and those relative frequencies p_{sample}. This process is repeated 10000 times to construct an approximate distribution of R-values which results from random sampling. From this distribution of R values, we determined a one-sided 95% confidence interval (i.e. the 95% quantile). The upper bound of this confidence interval marks the maximal R for a distribution which

cannot (significantly) be ruled out to stem from the same population as the experimental data. One shortcoming of this bootstrap method is that it cannot produce any values which have not been observed in experiment (e.g., $S = 1$) and, thus, does not take any error from these bins into account. Therefore, this method will underestimate the R confidence interval. To estimate errors for the $p[S] = 0$ bins, a second estimation has been made:

Beta-quantile method: For this method, we use the fact, that the number of observations of S synapses in experiment $N[S] := N_{exp} \cdot p_{exp}[S]$ can be considered as a binomially distributed random variable $N[S] \sim Bin(p_{real}[S], N_{exp})$ (the measured neuron pairs are independent and have S synapses with a certain probability $p_{real}[S]$). To estimate the errors of each histogram bin, we calculate confidence interval of the underlying probability $p_{real}[S]$ from the number of observations in experiment. For this, the quantiles of the beta function (a continuous pendant of the binomial distribution) can be used [82, 83]: We calculate the lower bound of the confidence interval as

$$p_L[S] = B^{-1}(\alpha/2, N_{exp}p_{exp}[S], N_{exp}(1 - p_{exp}[S]) + 1)$$

and the upper bound as

$$p_U[S] = B^{-1}(\alpha/2, N_{exp}p_{exp}[S] + 1, N_{exp}(1 - p_{exp}[S])) \ ,$$

where $B^{-1}(\alpha/2, a, b)$ gives the $\alpha/2$ quantile of the $Beta(a, b)$-distribution. As errors are normally depicted as one standard deviation for normal-distributed quantities, we use $\alpha = 33\%$.

From these errors, we can also calculate a maximum residual R_{single}, which corresponds to a distribution which has the maximal allowed error for each number of synapses

As an example, we calculated confidence intervals for the experimental distribution of cortical layer 4 to layer 4 connections in Figure S1A. The resulting errors indicate the significance of the two peaks, but allow for a broad range of different bimodal distribution shapes.

For comparison we also show the R values for the equilibrium distributions which were calculated in Figure 4. The maximal residual calculated as sum of the depicted error bars in Figure S1A is $R_{single} \approx 1.45$ (black contour Figure S1A). The upper bound of the 95% confidence interval from bootstrapping evaluated to $R = 1.04$ (white contour Figure S1B). Note, the activity region in which the equilibrium distributions of our model yield smaller R-values then the estimated error of the data are comparable to confidence regions of Monte-Carlo-p-value (grey contour Figure S1B). This means that, in the p-value confidence regions, our simple model yields reasonably low R-values without fine-tuning. However, the R-values are still bigger than those of previous works, which aim to fit the experimental data closely [43].

References

[82] Brown LD, Cai TT, DasGupta A (2001) Interval estimation for a binomial proportion. Statistical Science .

[83] Cameron E (2011) On the estimation of confidence intervals for binomial population proportions in astronomy: The simplicity and superiority of the bayesian approach. Publications of the Astronomical Society of Australia 28: 128-139.
