# Supplementary material for: The Formation of Multi-synaptic Connections by the Interaction of Synaptic and Structural Plasticity and Their Functional Consequences
Source: PLoS Comput Biol. 2015 Jan 15;11(1):e1004031. doi: 10.1371/journal.pcbi.1004031 (PMC4295841; doi:10.1371/journal.pcbi.1004031)
Supplement: Supporting Text S2 — (PDF) [file pcbi.1004031.s002.pdf]

Supporting Information for

The formation of multi-synaptic connections by the interaction of synaptic and structural plasticity and their functional consequences

Michael Fauth*, Florentin Wörgötter, Christian Tetzlaff
∗ E-mail: mfauth@gwdg.de

Derivation of the fixed-points of the different plasticity rules

The following section contains a short summary of the analysed synaptic plasticity rules and the derivation of their fixed points. The formulae for the fixed points can then be used to evaluate if the plasticity rule can lead to a positive $v_i^* - w_{ij}^*$ -dependence, which is the necessary condition for the emergence of biological realistic connectivity. As the neurons are usually situated in recurrent networks, we also calculate the fixed weights for a first-order approximation of a feedback system, where the presynaptic activity grows linearly with the postsynaptic activity: $v_j = v_i$. For all rules, we use a learning rate μ, the presynaptic activity v_j, the postsynaptic activity v_i, and the weight of the corresponding synapse w_{ij}.

Hebb or fixed-threshold BCM rule with hard boundaries: The Hebb rule $\dot{w}_{ij} = \mu v_j v_i$ exhibits only positive weight changes (as activities are positive) and will converge to the upper boundary w_{max}. For postsynaptic activities below a fixed LTP/LTD-threshold θ, the fixed BCM-rule $\dot{w}_{ij} = \mu v_j v_i (v_i - \theta)$ shows negative weight changes and converges to the lower boundary w_{min}, while for $v_i > \theta$ the weight changes are positive and the weight converges to the upper boundary w_{max}.

BCM rule with sliding threshold: For the original form of the BCM rule, the fixed θ is replaced by a multiple of the expected value of the squared postsynaptic activity: $\theta(t) = \tilde{\theta} E[v_i^2]$. For the average activities, this results in $\dot{w}_{ij} = \mu v_j v_i (v_i - \tilde{\theta} v_i^2)$. Setting $\dot{w}_{ij}$ to zero results in $0 = \mu v_j v_i^2 (1 - \tilde{\theta} v_i)$, which means that for non-zero presynaptic activities $v_i = 0$ or $v_i = \tilde{\theta}^{-1}$ lead to fixed weights. Given I_i leads to a baseline activity above zero and the weights cannot become negative, only the nonzero-activity fixed point remains. From inverting the input-output relation F of the neuron model one can derive the necessary weight to obtain this activity: $w_{ij}^* = \left(F^{-1}[\tilde{\theta}^{-1}] - I_i\right)/v_j$. As there is only one stable postsynaptic activity, there is also only one fixed weight and, thus, no continuous $v_i^* - w_{ij}^*$-dependence.

Using the feedback condition $v_j = v_i$, this results in a fixed weight $w_{ij}^* = \left(F^{-1}[\tilde{\theta}^{-1}] - I_i\right)/\tilde{\theta}^{-1}$ for the feedback-system. Again, there is only one stable postsynaptic activity, which yields no continuous $v_i^* - w_{ij}^*$-relation.

Oja rule: Setting the derivative $\dot{w}_{ij}$ to zero we obtain

$$0 = \mu v_i (v_j - w_{ij} v_i) \qquad \Rightarrow \qquad w_{ij}^* = v_j / v_i$$

which clearly shows a falling $v_i^* - w_{ij}^*$-dependence.

In the first order feedback system, where $v_j = v_i$, the fixed weight will be $w_{ij} = 1$, which cannot yield a positive $v_i^* - w_{ij}^*$-relation.

Up to this point, the results show that typically used rate based learning rules do not fulfil the necessary condition of our analysis. In the following, we analyse two rules including synaptic scaling - a homoeostatic mechanism which scales the weights up when the network activity is low and down when the network activity is high

Hebb rule and synaptic scaling: We will consider the equation $\dot{w}_{ij} = \mu(v_j v_i + \kappa^{-1}(v_{tss} - v_i) w_{ij}^n$. Setting this derivative to zero results to $\kappa v_j v_i = (v_i - v_{tss}) w_{ij}^{*n} \Rightarrow w_{ij}^* = \sqrt[n]{(v_j v_i)/(v_i - v_{tss})}$. The derivative

$$\frac{dw_{ij}^*}{dv_i} = \frac{1}{n} \left(\frac{v_j v_i}{v_i - v_{tss}} \right)^{(1-n)/n} \frac{-v_j v_{tss}}{(v_i - v_{tss})^2}$$

is negative for $v_i > v_{tss}$, which means that the weight will fall with the postsynaptic activity.

In the first order feedback system, the fixed weight results in $w^*_{ij} = \sqrt[n]{v_i^2/(v_i - v_{tss})}$. The derivative

$$\frac{dw^*_{ij}}{dv_i} = \frac{1}{n}\left(\frac{v_i^2}{v_i - v_{tss}}\right)^{(1-n)/n} \frac{v_i(v_i - 2v_{tss})}{(v_i - v_{tss})^2}$$

becomes positive for $v_i > 2v_{tss}$ which yields a growing $w^* - v^*$-relation. This will hold for all $n \geq 1$

BCM rule + synaptic scaling: When setting $\dot{w}_{ij}$ to zero for the learning rule $\dot{w}_{ij} = \mu(v_j v_i(v_i - \theta) + \kappa^{-1}(v_{tss} - v_i)w^n_{ij}$, we obtain $\kappa v_j v_i(v_i - \theta) = (v_i - v_{tss})w^{*n}_{ij}$ and, thus, $w^*_{ij} = \sqrt[n]{v_j v_i(v_i - \theta)/(v_i - v_{tss})}$. Because of the higher power of v_i in the numerator as compared to the denominator, the fixed weight clearly grows with v_i as long as $v_i > v_{tss}$ and $v_i > \theta$. This also holds true for the feedback case, where the fixed weight evaluates to $w^*_{ij} = \sqrt[n]{v_i^2(v_i - \theta)/(v_i - v_{tss})}$, such that this learning rule generates a growing $v^* - w^*$-relation in both feedforward and feedback case.

These results indicate that Hebbian or BCM-plasticity in combination with synaptic scaling fulfil the necessary condition, and, thus, that biological observed connectivity could emerge from weight-dependent stochastic structural plasticity in such systems.
