# Supplementary material for: The Formation of Multi-synaptic Connections by the Interaction of Synaptic and Structural Plasticity and Their Functional Consequences
Source: PLoS Comput Biol. 2015 Jan 15;11(1):e1004031. doi: 10.1371/journal.pcbi.1004031 (PMC4295841; doi:10.1371/journal.pcbi.1004031)
Supplement: Supporting Text S3 — We simulated the Input-Output relations of different neuron models to demonstrate that a concave Input-Output-relations is a common feature. (PDF) [file pcbi.1004031.s003.pdf]

Supporting Information for

The formation of multi-synaptic connections by the interaction of synaptic and structural plasticity and their functional consequences

Michael Fauth*, Florentin Wörgötter, Christian Tetzlaff
∗ E-mail: mfauth@gwdg.de

Input-Output-relations of different neuron models

The sufficient condition for a bimodal distribution of the number of synapses can be fulfilled by either a negatively curved weight-to-postsynaptic-activity relation or a negatively curved input-output relation for the neuron. Here we test whether the latter condition is fulfilled by biological reasonable neuron models. For this, we analysed the input-output-relations of several neuron models which perform well in approximating an experimentally observed spike-train evoked by a given current signal [84, 85]. To generate input-output-relations for those models, we simulated the output frequencies for varying input currents. Figure S2C-I shows that a negatively curved input-output relation can be fulfilled over a wide range of stimulations. Thus, in the corresponding frequency intervals, our sufficient condition can be fulfilled by biological reasonable neurons.

Note, stronger noise in the input to these models typically generates an interval with positive curvature at low frequencies. This feature is covered by the rate-based neuron models, which we show in the first two panels for comparison (Fig. S2A-B). When the system is assumed to work in these regimes, a sufficiently strong negative curvature of the $v_i^* - w_{ij}^*$-relation would be required as sufficient condition.

Used neuron models and parameters: Note, in the following models, the input or injected current into the neuron, which is the abscissa in all panels of Figure S2, will be denoted by I or I_{ext} .

A: Logistic function Output is given by

$$f = (1 + \exp(-I))^{-1}$$

B: Tangens hyperbolicus Output is given by

$$f = (\tanh(I) + 1)/2$$

C: Izhikevich neuron The Izhikevich-model [86] has 2 dynamic variables given by

$$\dot{v} = 0.04v^2 + 5v + 140 - u + I \quad \text{and} \quad \dot{u} = a(b \cdot v - u).$$

When $v > 30$ a spike is elicited and the dynamic variables are set to $v \leftarrow c$, $u \leftarrow u + d$. For regular firing neurons considered here, $a = 0.02, b = 0.2, c = -65$ and $d = 8$ are used. The amplitude of the injected current I was altered by up to $\pm 1\%$ of its initial strength at every time step (0.1 ms for Euler-method).

D-F: MAT-model The MAT model [87] has been shown to exhibit many of the behaviours of cortical spiking neurons [88] with the parameters used here. It consists of a membrane potential following $\dot{v} = -v/\tau_m + I$ ($\tau_m = 10$ ms) and a threshold which adapts on multiple time scales. The threshold for spiking is given as the sum of the different time scale contributions: $\theta = \sum_i \alpha_i \theta_i + \omega$ with $\dot{\theta}_i = -\theta_i/\tau_i$. Upon each spike, θ is increased by one and the neuron becomes refractive for a certain time interval (here 2 ms) but the membrane potential is not altered. For all parameter-sets shown here, two time scales $\tau_1 = 10$ ms and $\tau_2 = 100$ ms were used with different weightings:

Optimal	$\omega = 21, \alpha_1 = 180, \alpha_2 = 1$
Type I	$\omega = 5, \alpha_1 = 15, \alpha_2 = 3$
Type II	$\omega = 5, \alpha_1 = 15, \alpha_2 = -0.05$

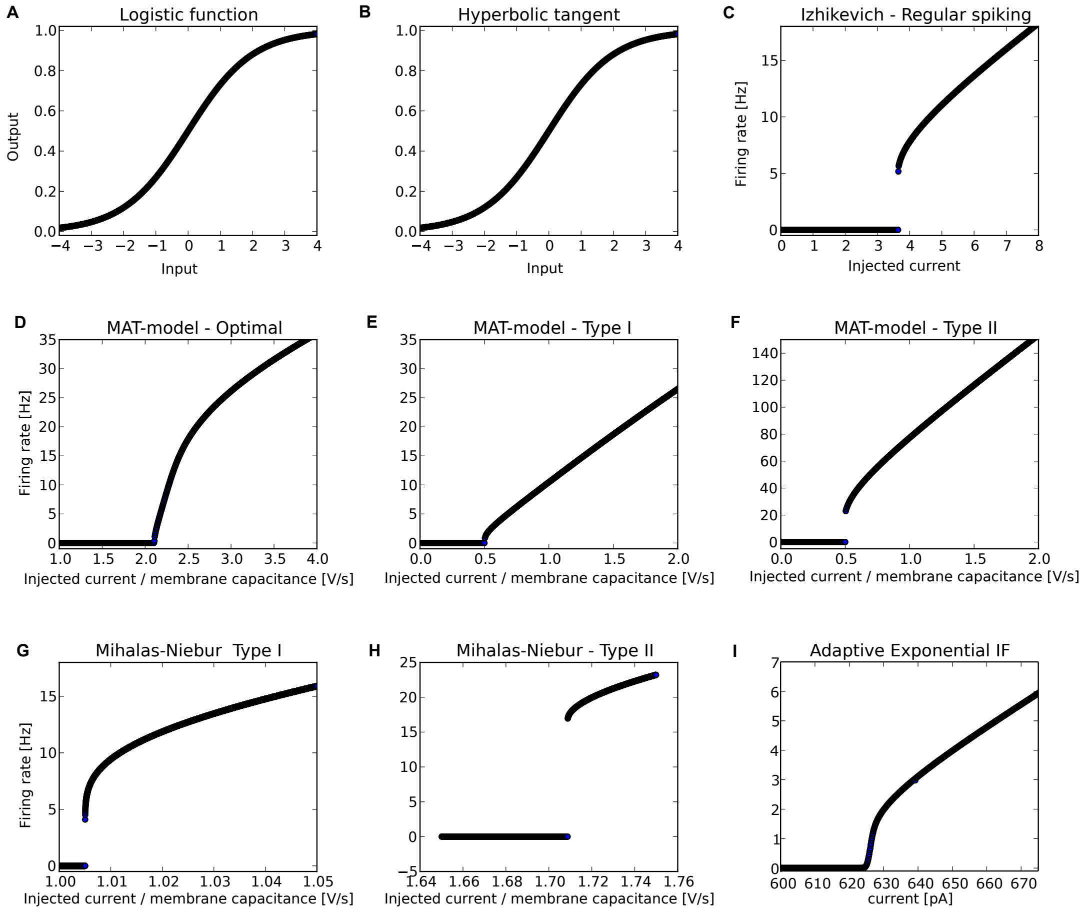

Supporting Figure S2. Input-Output-relations of different neuron models

Here "Optimal" denotes the parameter-set which performed best in explaining experimental spike times [89], and the "Type I / II" parameter-sets have been demonstrated to show the typical spiking behaviour of the corresponding neuron type. The injected current amplitude (in units of voltage change per time to spare a membrane capacitance parameter) was altered by 5% at every time step (0.1 ms for Euler-method).

G-H: Mihalas-Niebur model In this model [90], given an injected current I_{ext} (again given as I_{ext}/C in V/s), the membrane potential develops according to

$$\dot{v} = \frac{1}{C}\left(\sum_i I_i + I_{ext} - G(v - E_l)\right) \qquad \text{with internal currents } \dot{I}_i = -k_i I_i \quad i = 1...N.$$

The threshold for spiking is given by $\dot{\theta} = a(v - E_l) - b(\theta - \theta_\infty)$. When the membrane potential exceeds this threshold, the variables are reset to: $I_i \leftarrow R_i I_i + A_i$, $V \leftarrow V_r$ and $\theta = \max(\theta_r, \theta)$. For the shown simulations, we use two currents I_1 and I_2 with $b = 10$Hz, $G/C = 50$Hz, $k_1 = 200\text{s}^{-1}$, $k_2 = 20\text{s}^{-1}$, $R_1 = 0$, $R_2 = 1$, $\theta_\infty = 50$mV, $E_l = V_r = -70$mV and $\theta_r = -60$mV. As given in [90], we use $a = 0\ \text{s}^{-1}, A_1/C = A_2/C = 0\ \text{Vs}^{-1}$ for Type I and $a = 5\ \text{s}^{-1}, A_1/C = A_2/C = 0\ \text{Vs}^{-1}$ for Type II-neuron simulations.

I: Adaptive Exponential Integrate-and-Fire neuron The membrane-potential of this model [54] is given by

$$\dot{v} = \frac{1}{C}\left(-g_l(V - E_l) + g_l\Delta_T \exp\left(\frac{V - V_t}{\Delta_t}\right) - w + I\right)$$

with an adaptation current $\dot{w} = 1/\tau_w(a(v - E_l) - w)$. When v exceeds 30mV, it is reset to E_l and w is increased by $b = 0.0805$nA. For the simulations, we use $C = 281$pF, $g_l = 30$nS, $E_l = -70.6$mV, $V_T = -50.4$mV, $\Delta_T = 2$mV, $\tau_w = 144$ms, $a = 4$nS as given in [54].

References

[84] Jolivet R, Kobayashi R, Rauch A, Naud R, Shinomoto S, et al. (2008) A benchmark test for a quantitative assessment of simple neuron models. J Neurosci Methods 169: 417–424.

[85] Gerstner W, Naud R (2009) Neuroscience. How good are neuron models? Science 326: 379–380.

[86] Izhikevich EM (2003) Simple model of spiking neurons. IEEE Trans Neural Netw 14: 1569–1572.

[87] Kobayashi R, Tsubo Y, Shinomoto S (2009) Made-to-order spiking neuron model equipped with a multi-timescale adaptive threshold. Front Comput Neurosci 3: 9.

[88] Izhikevich EM, Gally JA, Edelman GM (2004) Spike-timing dynamics of neuronal groups. Cereb Cortex 14: 933–944.

[89] Yamauchi S, Kim H, Shinomoto S (2011) Elemental spiking neuron model for reproducing diverse firing patterns and predicting precise firing times. Front Comput Neurosci 5: 42.

[90] Mihalas S, Niebur E (2009) A generalized linear integrate-and-fire neural model produces diverse spiking behaviors. Neural Comput 21: 704–718.
