# Supplementary material for: The Formation of Multi-synaptic Connections by the Interaction of Synaptic and Structural Plasticity and Their Functional Consequences
Source: PLoS Comput Biol. 2015 Jan 15;11(1):e1004031. doi: 10.1371/journal.pcbi.1004031 (PMC4295841; doi:10.1371/journal.pcbi.1004031)
Supplement: Supporting Text S4 — For layer IV, the probability distributions from the experiment and the analysis with approximations are compared to a distribution obtained from a full simulation of the model dynamics. (PDF) [file pcbi.1004031.s004.pdf]

Supporting Information for

The formation of multi-synaptic connections by the interaction of synaptic and structural plasticity and their functional consequences

Michael Fauth*, Florentin Wörgötter, Christian Tetzlaff
* E-mail: mfauth@gwdg.de

Comparison of predicted distribution with simulations

To verify whether the approximations which have been used throughout our analysis are valid, we simulate the dynamics of a single connection considered in Figure 4 and evaluate the fraction of the simulated time it has a certain number of synapses. In the simulations, the differential equations have been implemented using a 4th order Runge-Kutta method. The learning rate was set to $\mu = 0.55$ and each simulation ran for $4,295 \cdot 10^9$ time steps. Figure S3 shows that the simulated distribution does not significantly differ from the approximation.

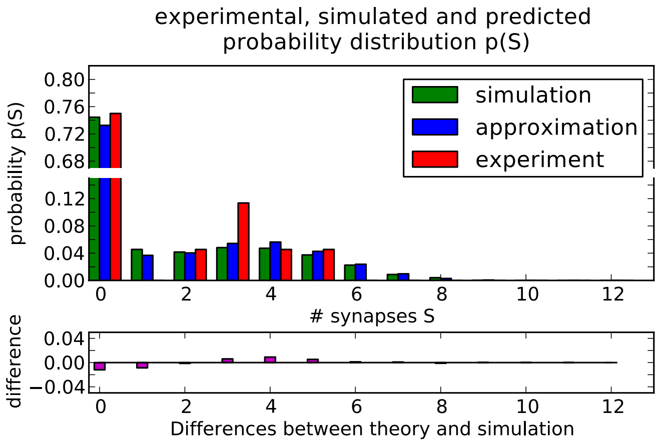

Supporting Figure S3. Comparison of the distributions emerging from simulation, analysis with approximation and experiment. For the system shown in Figure 4A, a simulation of the full dynamics without the first-step approximation yields a similar distribution as predicted by the approximation.
