# Supplementary material for: The Formation of Multi-synaptic Connections by the Interaction of Synaptic and Structural Plasticity and Their Functional Consequences
Source: PLoS Comput Biol. 2015 Jan 15;11(1):e1004031. doi: 10.1371/journal.pcbi.1004031 (PMC4295841; doi:10.1371/journal.pcbi.1004031)
Supplement: Supporting Text S5 — We show that the demonstrated effects do not strongly depend on the wij4/3-dependency in the deletion probability by repeating the analysis from Fig. 4 to 5 for a wij2-dependency. (PDF) [file pcbi.1004031.s005.pdf]

Supporting Information for

The formation of multi-synaptic connections by the interaction of synaptic and structural plasticity and their functional consequences

Michael Fauth*, Florentin Wörgötter, Christian Tetzlaff
* E-mail: mfauth@gwdg.de

Sensitivity to the power of w_{ij}

In this section, we show that the obtained results do not sensitively depend on the mathematical formulation of equation 1. Therefore, we repeated the calculations for our example neuron model and plasticity rule with a deletion probability given by

$$p_{del}[w_{ij}] = p_{build}^{\rho} \exp\left(-a^2 w_{ij}^2\right).$$

Supporting Figures S4-S6 show that the results are qualitatively the same as for the 4/3 exponent.

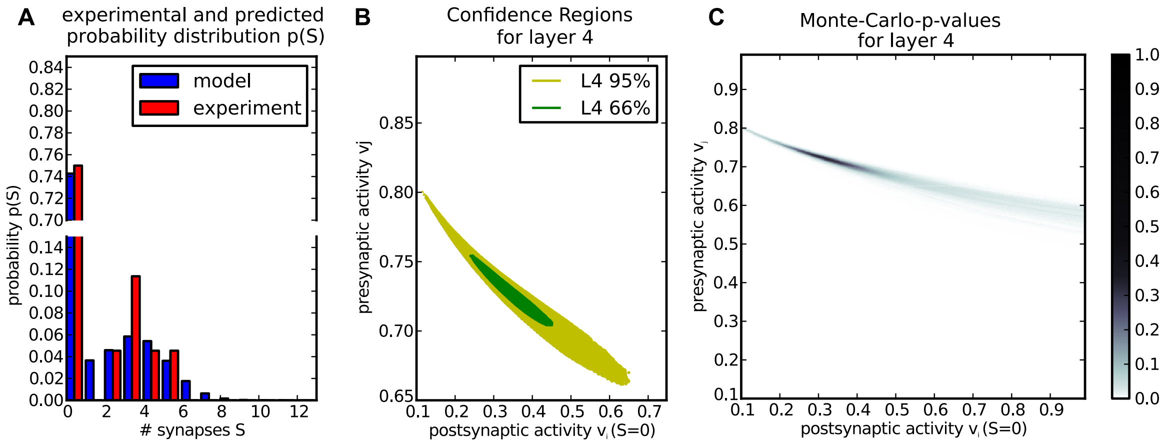

Supporting Figure S4. Model can account for experimental data for suitable pre- and postsynaptic activities. Same as Figure 4 but with $p_{del}[w_{ij}] = p^{\rho}_{build} \exp\left(-a^2 w^2_{ij}\right)$. (Parameters: BCM-rule with synaptic scaling with $\theta = 0.08, v_{tss} = 0.1, \kappa = 9.0$, structural plasticity: $P = 12, \ln p_{build} = -16, a = 1.4, \rho = 0.125$, in A: $v_j = 0.728, v_i(S = 0) = 0.3375$)

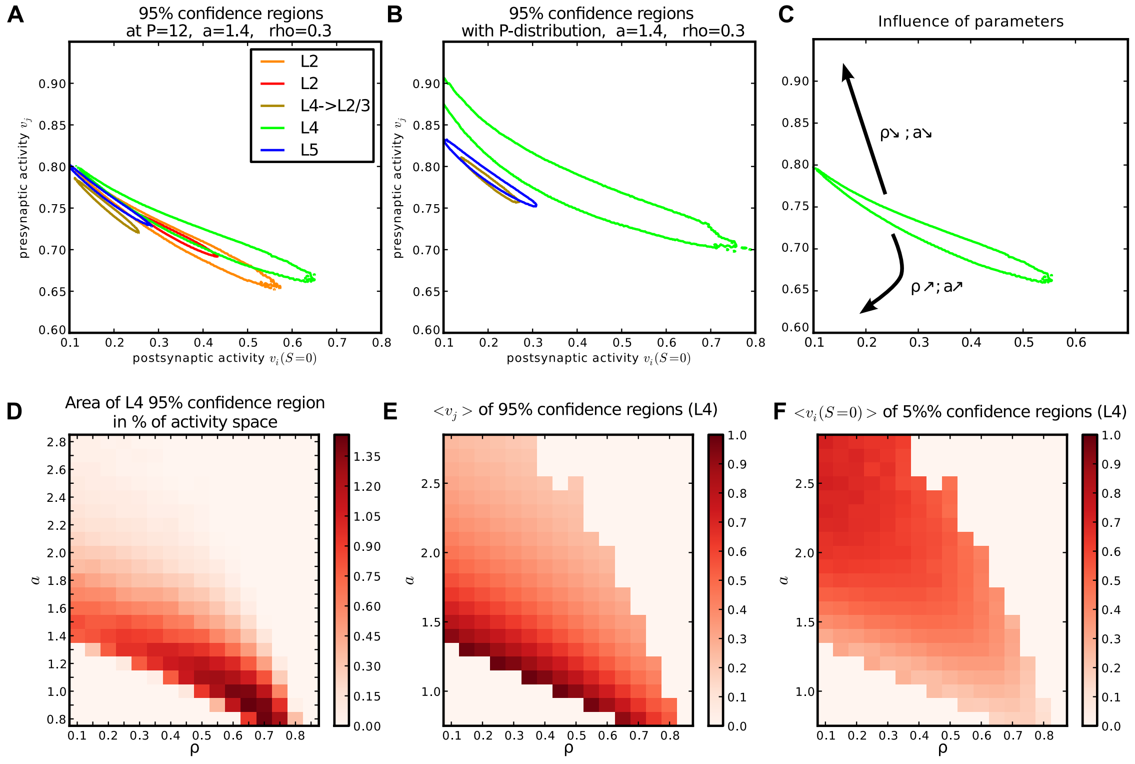

Supporting Figure S5. Different experimental data can be explained at different activity regions; effects are robust to underlying distributions or parameter changes. Same as Figure 5 but with $p_{del}[w_{ij}] = p^{\rho}_{build} \exp\left(-a^2 w^2_{ij}\right)$. (Parameters BCM-rule with synaptic scaling with $\theta = 0.08, v_{tss} = 0.1, \kappa = 9.0$, structural plasticity: $P = 12, \ln p_{build} = -16$, in A-C: $a = 1.4, \rho = 0.125$)

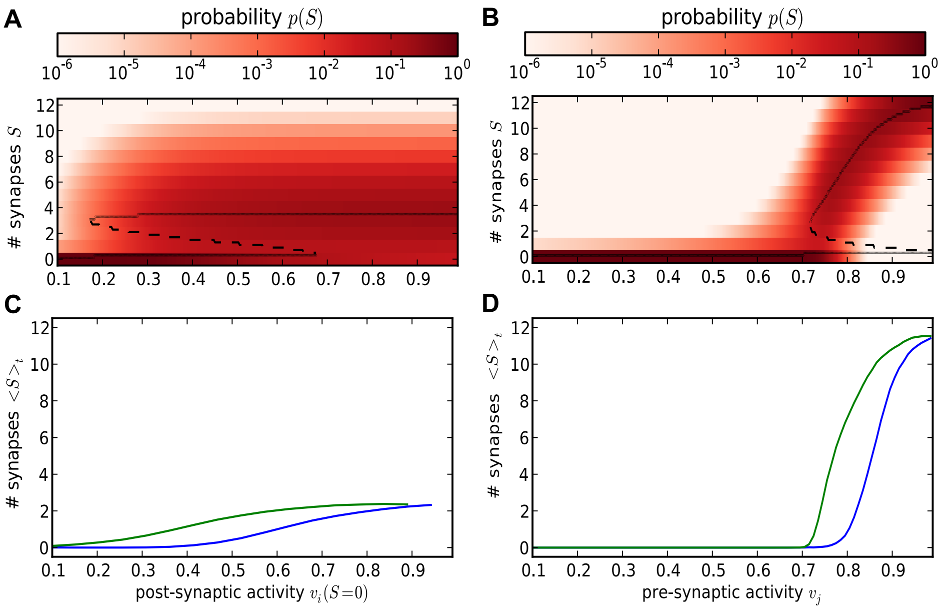

Supporting Figure S6. The BCM-rule feedforward connection shows a hysteresis in pre- and post-synaptic stimulation. Same as Figure 7 but with $p_{del}[w_{ij}] = p_{build}^{\rho} \exp\left(-a^2 w_{ij}^2\right)$. (Parameters: BCM-rule with synaptic scaling with $\mu = 0.2$, $\theta = 0.08, v_{tss} = 0.1, \kappa = 9.0$, structural plasticity: $P = 12, \ln p_{build} = -16, a = 1.4, \rho = 0.125$, in *A, C*: $v_j = 0.728$, 8811 cycles with $2 \cdot 10^6$ time steps per stimulation in steps of $\Delta v = 0.05$; in *B, D*: $v_i(S = 0) = 0.3375$) , 441 cycles with $6 \cdot 10^6$ time steps per stimulation
