# Supplementary material for: The Formation of Multi-synaptic Connections by the Interaction of Synaptic and Structural Plasticity and Their Functional Consequences
Source: PLoS Comput Biol. 2015 Jan 15;11(1):e1004031. doi: 10.1371/journal.pcbi.1004031 (PMC4295841; doi:10.1371/journal.pcbi.1004031)
Supplement: Supporting Text S6 — In this text the similarities and differences to the cascade model are discussed in greater detail. (PDF) [file pcbi.1004031.s006.pdf]

Supporting Information for

The formation of multi-synaptic connections by the interaction of synaptic and structural plasticity and their functional consequences

Michael Fauth*, Florentin Wörgötter, Christian Tetzlaff
* E-mail: mfauth@gwdg.de

Relation to cascade model

In the following we show the relation between the here presented model and the cascade model of synaptically stored memories ([72], see Fig. S7A). The latter model proposes synapses which have two functional states each consisting of a cascade of meta-plastic sub-states. During learning, a facilitating event may lead to a transition from the lower functional state into the first sub-state of the upper functional state (green arrows) or, if the system is already in that state, to a transition into the next deeper state of the upper cascade with certain probabilities (black arrows). The same applies for the depressing events and the lower functional state (red arrows). For both cases, the probability for a transition to the other functional state decreases with the depth in the cascade (ideally like an exponential decay). Thus, if all sub-states have a non-zero probability, the overall probability that the system changes from one functional state to the other is generated from the transition probabilities of all sub-states of the initial functional state, and, thus, by the interaction of a variety of Poisson processes (or exponential processes) on multiple time scales. The authors of the cascade model show that this interaction results in a power-law decay of the signal-to-noise ratio of patterns stored in a population of such synapses (memory) while learning new patterns. As we show in the following, our model exhibits a similar interaction of exponential processes, and, thus, should exhibit a power-law forgetting of structurally stored memories.

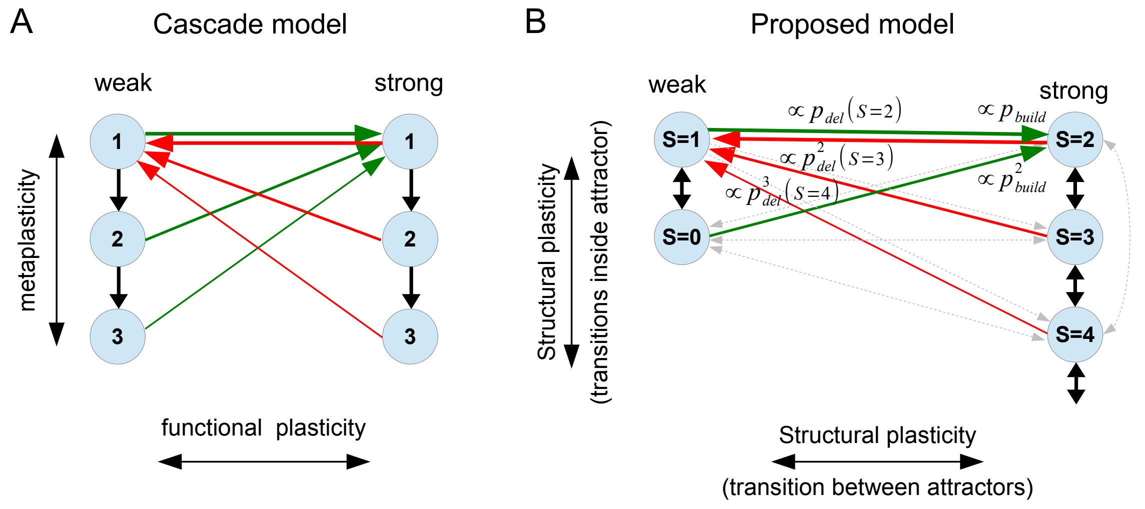

Supporting Figure S7. Structural plasticity with two attractors resembles cascade-model *(A)* scheme illustrating the cascade model (replotted and modified from [72]) *(B)* scheme of the here proposed model with the two basins of attraction as weakly and strongly connected states and the transitions corresponding to the cascade model highlighted in the same colors. Note, there are more transitions possible (light grey arrows) which might add further time scales.

The similarity between the two models reveals itself at the biological working point of our single connection, at which we observe two basins of attraction. These basins of attraction can be interpreted as the functional states and the actual number of synapses as the sub-states. Thus, transitions inside one basin of attraction correspond to metaplasticity, while transitions from one attractor to another correspond to functional plasticity in the cascade model. Note, each of the sub-states of one basin of attraction in our model has a different weight and will lead to different activity dynamics. However, as the analysis of the cascade model does not rely on activity dynamics, this difference can be neglected for the structural analogy.

Furthermore, the sub-states in our model are not arranged as a cascade, but allow for transitions in both directions. Nevertheless, all sub-states are populated with sufficiently high probabilities, because synaptic plasticity adjusts the deletion probabilities accordingly. At each of these sub-state there is also a probability to build or remove enough synapses to change the system's basin of attraction (functional state). These probabilities also vary over many orders of magnitude (e.g., $p^1_{del}, p^2_{del}, ...$) depending on how many synapses have to be build or removed to transit to the other basin of attraction. Thus, the transition to the other basin of attraction is composed of multiple exponential processes at different time scales. Transitions which target states deeper inside the other basin of attraction could add even more and longer time scales to that. Thus, we expect that the collective dynamics of all synapses on one connection between two neurons is similar to the power-law forgetting in the cascade model, but on a much slower time scale.
