# Supplementary material for: The Formation of Multi-synaptic Connections by the Interaction of Synaptic and Structural Plasticity and Their Functional Consequences
Source: PLoS Comput Biol. 2015 Jan 15;11(1):e1004031. doi: 10.1371/journal.pcbi.1004031 (PMC4295841; doi:10.1371/journal.pcbi.1004031)
Supplement: Supporting Text S7 — This text describes how the calcium based plasticity rule [24] is analytically integrated for the simulations. (PDF) [file pcbi.1004031.s007.pdf]

Supporting Information for

The formation of multi-synaptic connections by the interaction of synaptic and structural plasticity and their functional consequences

Michael Fauth*, Florentin Wörgötter, Christian Tetzlaff
* E-mail: mfauth@gwdg.de

Integration of the Graupner-Brunel plasticity rule

Consider the Graupner-Brunel calcium-based plasticity rule without the bistable potential term [24]:

$$\begin{aligned} \tau_w \frac{dw(t)}{dt} &= \gamma_p(1-w(t))\Theta[c(t)-\theta_p] - \gamma_d w \Theta[c(t)-\theta_d] \\ \frac{dc(t)}{dt} &= -c(t)/\tau_c + \sum_k C_{\text{pre}}\delta(t-t_{pre,k}) + \sum_l C_{\text{post}}\delta(t-t_{post,l}) \quad , \end{aligned}$$

where Θ denotes the Heavyside-stepfunction and $t_{pre,k}$ ans $t_{post,l}$ are the times of the kth presynaptic spike and the lth postsynaptic spike. Given the last spike occurred at $\tilde{t}$, the time evolution of c is given by:

$$c(t) = c(\tilde{t})e^{-(t-\tilde{t})/\tau_c}.$$

The evolution of w is governed by three different equations depending on the value of $c(t)$. For $\theta_p > \theta_d$ the following three steps describe how to calculate the resulting weight w and calcium level c at the next spike time t_{next} when successively applied:

1. when $c(\tilde{t}) > \theta_p$ (LTP-phase):

$$\begin{aligned} t_1 &\leftarrow \min(\tilde{t} - \tau_c * \log(\theta_p/c(\tilde{t})), t_{next}) \\ w &\leftarrow \frac{\gamma_p}{\gamma_p+\gamma_d} - \left(\frac{\gamma_p}{\gamma_p+\gamma_d} - w\right) e^{-(\gamma_p+\gamma_d)\cdot(t_1-\tilde{t})/\tau_w} \\ c(t_1) &\leftarrow c(\tilde{t})e^{-(t_1-\tilde{t})/\tau_c} \end{aligned}$$

   else $t_1 \leftarrow \tilde{t}$, $c(t_1) \leftarrow c(\tilde{t})$

2. when $t_1 < t_{next}$ and $c(t_1) > \theta_d$ (LTD-phase):

$$\begin{aligned} t_2 &\leftarrow \min(t_1 - \tau_c * \log(\theta_d/c(t_1)), t_{next}) \\ w &\leftarrow w \cdot \exp(-\gamma_d \cdot (t_2 - t_1)/\tau_\rho) \\ c(t_2) &\leftarrow c(t_1)e^{-(t_2-t_1)/\tau_c} \end{aligned}$$

   else $t_2 \leftarrow t_1$, $c(t_2) \leftarrow c(t_1)$

3. when $t_2 < t_{next}$: (no change in w)

$$c(t_{next}) \leftarrow c(t_2)e^{-(t_{next}-t_2)/\tau_c}$$
