# Supplementary material for: The Formation of Multi-synaptic Connections by the Interaction of Synaptic and Structural Plasticity and Their Functional Consequences
Source: PLoS Comput Biol. 2015 Jan 15;11(1):e1004031. doi: 10.1371/journal.pcbi.1004031 (PMC4295841; doi:10.1371/journal.pcbi.1004031)
Supplement: Supporting Table S1 — For a broad variety of learning rules this table indicates whether the fixed weights fulfil the necessary or the sufficient condition. (PDF) [file pcbi.1004031.s008.pdf]

According to [21] rate based learning rules can be approximated by a Taylor series around zero by:

$$\dot{w}_{ij} \overset{T}{=} c_0[w_{ij}] + c_{1,pre}[w_{ij}]v_j + c_{2,pre}[w_{ij}]v_j^2 + c_{1,post}[w_{ij}]v_i + c_{2,post}[w_{ij}]v_i^2 + c_{corr}[w_{ij}]v_iv_j + \mathcal{O}[v^3].$$

The following table shows whether learning rules composed from several terms of this series fulfil the sufficient or necessary condition in the feedforward or feedback system. We assumed $v_i, v_j > 0$ and restricted our analysis to reasonable rules with stable fixed points. Furthermore, we added the rules shown in Figure 3.

**rule**	**differential equation** $\dot{w}_{ij}$	**feedforward** w^*_{ij}	necc. cond.	suff. cond.	**feedback** w^*_{ij} $(v_i = v_j)$	necc. con.	sufficient condition	**nonzero coefficients**
Hebb with hard boundaries	$\dot{w}_{ij} = \mu v_j v_i$ with $w_{ij} \in [w_{min}, w_{max}]$	w_{max}	-	-	w_{max}	-	-	c^{const}_{corr}
Hebb rule with decay	$\dot{w}_{ij} = \mu v_j v_i - \gamma w_{ij}$	$\mu/\gamma \cdot v_i v_j$	+	-	$\mu/\gamma \cdot v_i^2$	+	-	$c^{const}_{corr}, c_0(w_{ij})$
Hebb-rule with presynaptic-gated decay	$\dot{w}_{ij} = \mu v_j(v_i - \gamma w_{ij})$	v_i/γ	+	-	v_i/γ	+	-	$c^{const}_{corr}, c_{1,pre}(w_{ij})$
Hebb-rule with postsynaptic-gated decay	$\dot{w}_{ij} = \mu v_i(v_j - \gamma w_{ij})$	v_j/γ	-	-	v_i/γ	+	-	$c^{const}_{corr}, c_{1,post}(w_{ij})$
Hebb-rule with weight-dependent scaling $(n \geq 1)$	$\dot{w}_{ij} = \mu v_j v_i - \mu\kappa^{-1}(v_i - v_{tss})w^n_{ij}$	$\sqrt[n]{\frac{\kappa v_i v_j}{v_i - v_{tss}}}$	-	-	$\sqrt[n]{\frac{\kappa v_i^2}{v_i - v_{tss}}}$	$v_i > v_{tss}$	-	$c^{const}_{corr}, c_{1,post}(w_{ij}), c_0[w_{ij}]$
Hebb-rule with soft boundaries	$\dot{w}_{ij} = \mu(1 - w_{ij})v_j v_i$	1	-	-	1	-	-	$c_{corr}[w_{ij}]$
Hebb-rule with soft boundaries and decay	$\dot{w}_{ij} = \mu(1 - w_{ij})v_j v_i - \gamma w_{ij}$	$v_i v_j/(\gamma/\mu + v_i v_j)$	+	+	$v_i^2/(\gamma/\mu + v_i^2)$	+	$v_i > \sqrt{\frac{\gamma}{3\mu}}$	$c_{corr}[w_{ij}], c_0[w_{ij}]$
Hebb-rule with postsynaptic-gated decay and soft boundaries	$\dot{w}_{ij} = \mu v_i(v_j(1 - w_{ij}) - \gamma w_{ij})$	$v_j/(\gamma + v_j)$	-	-	$v_i/(\gamma + v_i)$	+	+	$c_{corr}[w_{ij}], c_{1,post}[w_{ij}]$
Hebb-rule with presynaptic-gated decay and soft boundaries	$\dot{w}_{ij} = \mu v_j(v_i(1 - w_{ij}) - \gamma w_{ij})$	$v_i/(\gamma + v_i)$	+	+	$v_i/(\gamma + v_i)$	+	+	$c_{corr}[w_{ij}], c_{1,pre}[w_{ij}]$
Oja-rule	$\dot{w}_{ij} = \mu(v_j v_i - w_{ij} v_i^2)$	$\frac{v_j}{v_i}$	-	-	1	-	-	$c^{const}_{corr}, c_{2,post}[w_{ij}]$
Bienenstock-Cooper-Munro-rule	$\dot{w}_{ij} = \mu \cdot v_j v_i(v_i - \theta)$							
... with fixed-θ and hard boundaries	with $w_{ij} \in [0, w_{max}]$	$w_{min};\ w_{max}$	-	-	$w_{min};\ w_{max}$	-	-	c^{const}_{corr}, higher
... with sliding threshold $\theta = \theta[t]$	fixed point: $\theta \to \tilde{\theta} \cdot {v_i^*}^2$	see Text S2	-	-	see Text S2	-	-	c^{const}_{corr}, higher order
fixed-threshold BCM-rule with weight-dependent scaling $(n > 1)$	$\dot{w}_{ij} = \mu v_j v_i(v_i - \theta) - \mu\kappa^{-1}(v_i - v_{tss})w^n_{ij}$	$\sqrt[n]{\frac{\kappa v_j v_i(v_i - \theta)}{v_i - v_{tss}}}$	$v_i > v_{tss}$ $v_i > \theta$	-	$\sqrt[n]{\frac{\kappa v_i^2(v_i - \theta)}{v_i - v_{tss}}}$	$v_i > v_{tss}$ $v_i > \theta$	-	c^{const}_{corr}, higher order
Graupner-Brunel rule	Spike-timing-dependent (see Methods)	no analytical solution	+	+	no analytical solution	+	+	-

+ ... always fulfilled, - ... never fulfilled, otherwise: interval where fulfilled. $c^{const}_{corr} \equiv c_{corr}[w_{ij}] = const$

Note, for simplicity the soft boundary has been set equal to 1.
